# Nonlinear dynamics and bifurcation structure of ultrasonically excited lipid coated microbubbles

**DOI:** 10.1016/j.ultsonch.2020.105405

**Published:** 2020-12-08

**Authors:** A.J. Sojahrood, H. Haghi, R. Karshafian, M.C. Kolios

**Affiliations:** a Department of Physics, Ryerson University, Toronto, Canada; b Institute for Biomedical Engineering, Science and Technology (IBEST) a partnership between Ryerson University and St. Michael’s Hospital, Toronto, Ontario, Canada

## Abstract

In many applications, microbubbles (MBs) are encapsulated by a lipid coating to increase their stability. However, the complex behavior of the lipid coating including buckling and rupture sophisticates the dynamics of the MBs and as a result the dynamics of the lipid coated MBs (LCMBs) are not well understood. Here, we investigate the nonlinear behavior of the LCMBs by analyzing their bifurcation structure as a function of acoustic pressure. We show that, the LC can enhance the generation of period 2 (P2), P3, higher order subharmonics (SH), superharmonics and chaos at very low excitation pressures (e.g. 1 kPa). For LCMBs sonicated by their SH resonance frequency and in line with experimental observations with increasing pressure, P2 oscillations exhibit three stages: generation at low acoustic pressures, disappearance and re-generation. Within non-destructive oscillation regimes and by pressure amplitude increase, LCMBs can also exhibit two saddle node (SN) bifurcations resulting in possible abrupt enhancement of the scattered pressure. The first SN resembles the pressure dependent resonance phenomenon in uncoated MBs and the second SN resembles the pressure dependent SH resonance. Depending on the initial surface tension of the LCMBs, the nonlinear behavior may also be suppressed for a wide range of excitation pressures.

## Highlights

• Bifurcation structure of the lipid coated microbubbles (MBs) is studied
• Effect of the coating is analyzed by comparing the dynamics of the lipid coated MBs to uncoated ones.
• Buckling and rupture of the lipid coating enhances the nonlinear oscillations.
• Period 2, Period 3 or chaos can be generated at pressures as low as 1 kPa.
• In addition to subharmonic emissions, superharmonic emissions may also be enhanced.
• With increasing pressure, period 2 is generated, then disappears and is regenerated again.

## 1 Introduction

A microbubble (MB) excited by an ultrasound pressure wave is an instance of a complex nonlinear dynamical system with resonances, several attractors and their basins, multiple bifurcations and chaotic behavior and not “yet fully describable behavior” due to its infinite complexity [1], [2], [3]. In spite of the complexity, MBs are used in industrial applications like cleaning [4], [5], food production [6], sonochemistry [7], [8], [9], sonoluminescence [9], [10], mixing [11], [12], therapeutic [13], [14], [15], [16] and diagnostic [17], [18], [19], [20] ultrasound.

One of the first nonlinear phenomena detected with MBs in sound fields was through historical observations of Esche [21]. Esche reported the generation of a frequency peak at half the excitation frequency (f) in the power spectrum of the received signal [21]. In his investigation of MBs driven with 3Hz-3.3 MHz, he found the appearance of spectral lines at f/2 and in some cases f/3 for sufficiently high acoustic pressures. In a continuation of Esche’s work, Bohn reported spectral lines down to f/4 [22]. In the chaotic (broadband noise) region of the sound emitted by the MBs, Holzfuss & Lauterborn [3] observed a surprisingly low-dimensional attractor with correlation dimension of about 2.5 which is the characteristic for driven damped nonlinear oscillators. Several other experimental studies investigated the nonlinear dynamics of ultrasonically excited MBs [23], [24], [25], [26], [27], [28], [29]; observing subharmonics, ultraharmonics and chaotic behavior. Numerical investigations have demonstrated the existence of multiple resonance peaks [2], [30], [31], period doubling route to chaos [32], [33], [34], strange attractors and chaotic behavior (e.g. [1], [2], [3], [28], [29]).

Within the last decade several studies have employed the methods of dynamical systems to study the behavior of MBs. There have been successful attempts in classification of some of the nonlinear dynamics of the MB oscillator [35], [36], [37], [38]. Hegedüs [39] found numerical evidence for the existence of stable period 1 solutions beyond Blake’s threshold [39]. Occurrence of higher order subharmonics (SHs) (f/3, f/4, f/5 etc) has been extensively investigated in [38], [40] and for the case of ambient pressures slightly below the vapor pressure [39]. They are experimentally observed and numerically modelled in [41], [42], [43].

Hegedus [44] studied the topology of stable periodic solutions near Blake’s threshold. The effect of high dissipation on the nonlinear evolution of the MB behavior is considered in [45], [46] and it has been shown that MB becomes an over-damped oscillator suppressing collapse-like behavior. Moreover, they reported the existence of transient chaos [45]. Using two frequencies was proposed in [47] and extended in [48], [49], [50], [51] to control the chaotic behavior of the MBs. The effect of multiple frequencies on the resonance behavior and nonlinear dynamics of the system was investigated in [52], [53], [54], [55].

The influence of the pressure amplitude on the resonance frequency and bifurcation structure of the MBs which is driven by its resonance frequency is studied in [56]. It is shown that increasing the incident ultrasound pressure decreases the resonance frequency of the MB; when the MB is sonicated with its pressure dependent resonance frequency a saddle node bifurcation takes place at the corresponding pressure amplitude which enhances the nondestructive back-scattered pressure by the MBs. Non-spherical MB oscillations in a viscous liquid is studied in detail in [46] and its been shown that the increased rate of dissipation can significantly extend the stable domains in the acoustic excitation parameter planes. We have studied the ultraharmonic (UH) and super harmonic (SuH) behavior of the MB oscillator by introducing a more comprehensive method of construction of bifurcation diagrams [57]. Using this method, the bifurcation structure of the MBs undergoing period doubling and 1/2 order sub-harmonic emissions have been extensively studied [34]. It was found that sonication of MBs with twice their linear resonance frequency results in period doubling at a lower excitation and leads to non-destructive stable period 2 oscillations, however, sonication with resonance will most likely result in MB destruction before the appearance of period 2 oscillations. We showed in [58] that SH resonance frequency decreases with increasing pressure; and maximum SH strength is generated when the sonication frequency is 1.5–1.6 times the resonance frequency of the MBs.

In spite of numerous studies on the complex behavior of free (uncoated) MBs, the dynamics of the coated MBs have not been thoroughly studied. MBs stabilized by a coating in the form of phospho-lipid (e.g. Definity®[59]), or albumin (e.g. Optison [60]) or polymer (Point [61]) are designed to be used in clinical and pre-clinical medical ultrasound applications. Addition of the coating (more specifically in case of phospho-lipid coating) immensely increases the complexity of the MB oscillator. During MB oscillations phospho-lipid shell can undergo buckling and rupture [62] resulting in a dynamical system with varying stiffness. The dynamic stiffness of the nonlinear oscillator enhances the generation of nonlinear signatures in the oscillation of the coated MBs.

Buckling of the lipid shell has been shown to be one of the possible reasons for enhanced non-linearity [62], [63], [64], [65], [66], [67], [68], [69], [70], [71]. Phospho-lipid shell MBs exhibit compression only behavior [67] during which MBs compress more than they expand. There exists a threshold behavior for the onset of oscillations [72]; the MB starts to oscillate only above a pressure threshold. It has been experimentally observed that phospholipid shell MBs can generate SH oscillations even at very low acoustic pressures (<30 KPa [64], [66], [73]). Such low threshold values not only contradict the predictions of the theoretical models for coated MBs [65], [74], [75], they are even below the threshold values expected for uncoated free MBs [65], [76]. The low pressure thresholds are despite the increased damping due to the presence of the shell. Through experiments and numerical simulations it has been shown in [64] that the low pressure threshold for SH emissions is due to the compression only behavior of the MBs due to the buckling of the shell.

In [68] the lipid shell was found to enhance the nonlinear MB response at acoustic pressures as low as 10 kPa. The increase in acoustic pressure amplitude lead to a substantial decrease of the frequency of the maximum response even at very low acoustic pressures [68] resulting in a pronounced skewness of the resonance curve. Such shift in resonance has been postulated in [68] to be the origin of the ’thresholding’ behavior [72]. Nonlinear resonance behavior of the lipid shell MBs was also observed in higher frequencies (5–15 MHz) in [77]. It is shown in [66] that the shell elasticity of the phospholipid shell varies with MB oscillation amplitude and the magnitude of ’compression only’ behavior depends on the initial phospholipid concentration on the MB surface. Prosperetti [65] through theoretical analysis of the Marmottant model [62] attributed the lower SH threshold of the lipid MBs to the variation in the mechanical properties of the shell in the neighborhood of a certain MB radius (e.g. the occurrence of buckling).

In addition to the widely studied 1/2 order SHs, we have experimentally detected higher order SHs (1/3, 1/4 and 1/5) in the oscillations of lipid coated MBs at very low acoustic pressures and high frequencies (e.g. 25 MHz) [41], [42], [43]. Through analyzing bifurcation diagrams we concluded that buckling or rupture of the shell is responsible for the enhanced nonlinear behavior [41], [42], [43]. The closer the initial surface tension of the MB to the two limit values of the buckling and rupture of the shell, the lower the pressure threshold for nonlinear oscillations. Variation of the mechanical properties of the shell can also manifest itself in expansion dominated behavior in liposome-loaded lipid shells [69]. Expansion dominated oscillations occur for MBs with an initial surface tension near that of water [69], [77]. Upon expansion, the stiffness of the coating weakens and the MB expands more than it compresses. Expansion-dominated behavior was used to explain the enhanced non-linearity at higher frequencies (25 MHz) [78]. The Marmottant model effectively captures the behavior of the MB including expansion-dominated behavior [59], [43], [77], compression only behavior [67], thresholding [72] and enhanced non-linear oscillations at low excitation pressures [41], [43], [63], [64], [66], [68].

Previous studies (e.g. [2], [34], [36], [38], [39], [40], [56], [58]) investigated the bifurcation structure of the uncoated bubbles and bubbles coated with shells that exhibit linear viscoelastic behavior. However, the influence of the nonlinear viscoelastic behavior of the coating (e.g. buckling and rupture [59], [62]) on the bifurcation structure of the bubble has not been investigated before. Due to the enhanced nonlinearity created by the behavior of the shell, it is important to rigorously investigate the impact of the exposure parameters on the MB oscillations. The current work addresses this problem for the first time. We perform a comprehensive analysis of the bifurcation structure of ultrasonically excited lipid coated MBs. Similar to our previous works in [34], [38], [56], [58] we study the radius vs excitation pressure amplitude bifurcation structure of the lipid coated bubbles when the bubble is sonicated with multiples of its resonance frequency. Results are then compared to previous studies whereby we reveal the influence of the nonlinear shell viscoelasticity on the bubble behavior. We show that the buckling and/or rupture of the shell enhances the subharmonics (SHs), superharmonics (SuHs), ultraharmonics (UHs) and chaos at very low excitation pressures. The enhanced non-linearity may disappear at moderate pressures. At higher pressures, nonlinear behavior may reappear in the bubble behavior exhibiting similar behavior to the uncoated bubbles and coated bubbles with linear viscoelastic behavior.

Knowledge of the effect of the shell behavior on the nonlinear response of the MB is essential to optimize the MBs response to an ultrasonic field. Moreover, the comprehensive knowledge that can be obtained through analyzing the bifurcation diagrams of the lipid coated MBs may help in revealing potential parameter spaces in which MB behavior can be beneficial to various applications. Last but not least, revealing the intricate behavior of the system and enhanced nonlinear effects is of potential interest in the field of nonlinear and chaotic dynamical systems.

## 2 Methods

### 2.1 Marmottant model

The dynamics of the coated MBs undergoing buckling and rupture can be effectively modeled using the Marmottant equation [62]:

(1) $$\begin{array}{c} \rho \left(R\overset{¨}{R}+\frac{3}{2}{\overset{˙}{R}}^{2}\right)=\\ \left[P_{0}+\frac{2\sigma (R_{0})}{R_{0}}\right]{\left(\frac{R}{R_{0}}\right)}^{-3k}\left(1-\frac{3k}{c}\overset{˙}{R}\right)-P_{0}-\frac{2\sigma (R)}{R}-\frac{4\mu_{L}\overset{˙}{R}}{R^{2}}-\frac{4k_{s}\overset{˙}{R}}{R^{2}}-P_{a}(t) \end{array}$$

In this equation, R is the bubble radius at time t, $R_{0}$ is the initial bubble radius, $\overset{˙}{R}$ is the wall velocity of the bubble, $\overset{¨}{R}$ is the wall acceleration, ρ is the liquid density (998 $\frac{\mathrm{kg}}{m^{3}}$), c is the sound speed (1481 m/s), $P_{0}$ is the atmospheric pressure, σ(R) is the surface tension at radius R, $\mu_{L}$ is the liquid viscosity (0.001 Pa s), $k_{s}$ is the viscosity of the coating, *k* is the polytropic index for the gas and $P_{a}(t)$ is the acoustic driving force $P_{a}(t)=P_{a}\sin(2\pi \mathrm{ft})$ where $P_{a}$ and *f* are the amplitude and frequency of the applied acoustic pressure. The values in the parentheses are for pure water at 293^0^K. In this paper the gas inside the bubble is C3F8 and water is the host media.

**Fig. 1:**
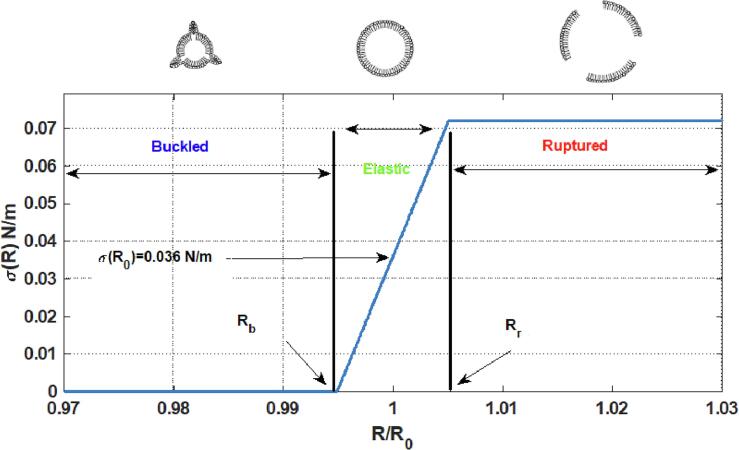
Schematic of the effective surface tension on a coated MB with $R_{0}=2\;\mu m,\chi =3.5\;N/m$ & $\sigma_{0}=0.036\;N/m$. The coating buckles when $R⩽R_{b}$ making the surface tension zero. The coating behaves elastically for $R_{b}⩽R⩽R_{r}$. When $R⩾R_{r}$, the coating ruptures and exposes the gas to water, thus the effective surface tension becomes equal to $\sigma_{\mathrm{water}}$ (0.072 N/m).

The surface tension σ(R) is a function of radius and is given by:

(2) $$\sigma (R)=\left\{\begin{array}{ccc} 0 & \mathrm{if} & R⩽R_{(}b)\\ \chi (\frac{R^{2}}{R_{b}^{2}}-1) & \mathrm{if} & R_{b}⩽R⩽R_{r}\\ \sigma_{\mathrm{water}} & \mathrm{if} & \mathrm{RupturedR}⩾R_{r} \end{array}\right)$$

where $\sigma_{0}$ is the initial surface tension (at $R=R_{0}$), $\sigma_{\mathrm{water}}$ is the water surface tension and χ is the shell elasticity. $R_{r}$ and $R_{b}$ are the rupture and the buckling radius respectively where $R_{b}=\frac{R_{0}}{\sqrt{1+\frac{\sigma (R_{0})}{\chi }}}$ and $R_{r}=R_{b}\sqrt{1+\frac{\sigma_{\mathrm{rupture}}}{\chi }}$. In this work similar to [78], $R_{\mathrm{breakup}}=R_{r}$. In this paper, simulations were run for different values of $\sigma_{0}$. The initial surface tension $\sigma_{0}$ is a property of the lipid coated bubble and varies when using different manufacturing methods [79], [80]. Moreover, $\sigma_{0}$ can be altered by varying the ambient pressure in the liquid [64], [80]. Variations in $\sigma_{0}$ changes the $R_{b}$ and $R_{r}$ which in turn change the dynamical behavior of the bubble. In this paper, for simplicity, and similar to [78], [80] we have assumed $\sigma_{\mathrm{rupture}}=\sigma_{\mathrm{water}}$. Fig. 1 shows a representation of buckling and rupture and the dependence of the effective surface tension (σ(R)) on microbubble radius.

### 2.2 Keller-Miksis model

Dynamics of the uncoated bubbles were also visualized alongside the lipid coated bubbles to highlight the effect of the lipid shell on the bubble dynamics. To model the uncoated bubble dynamics the Keller-Miksis model [79] is used:

(3) $$\rho \left[\left(1-\frac{\overset{˙}{R}}{c}\right)R\overset{¨}{R}+\frac{3}{2}{\overset{˙}{R}}^{2}\left(1-\frac{\overset{˙}{R}}{3c}\right)\right]=\left(1+\frac{\overset{˙}{R}}{c}\right)(G)+\frac{R}{c}\frac{d}{\mathrm{dt}}(G)$$

where $G=\left(P_{0}+\frac{2\sigma_{\mathrm{water}}}{R_{0}}\right)\;{\left(\frac{R}{R_{0}}\right)}^{-3k}-\frac{4\mu_{L}\overset{˙}{R}}{R}-\frac{2\sigma }{R}-P_{0}-P_{a}\sin(2\pi \mathrm{ft})$.

In both models we have neglected the effects of thermal damping. This is to decrease the problem complexity and to better highlight only the shell effects. Moreover, we have shown in [89] that in case of C3F8 gas cores thermal damping is significantly smaller compared to air. Moreover, in case of coated bubbles with C3F8 gas cores, thermal effects maybe be fully neglected. However, in case of the uncoated bubble effects of thermal damping at higher pressures should be considered using full ODEs [90] that account for the thermal damping. We have shown in [89] that the generally used linear assumptions [91] for thermal effects may lead to inaccuracies at pressures as low as ≈40kPa. However, since the main focus of the paper is to highlight the coating effects and because the thermal effects of the C3F8 are weak [88], we have neglected the thermal effects in this paper.

It should be noted that the Keller-Miksis model (Eq. (3)) has some additional terms compared to the the Marmottant model (Eq. (1)). In this paper, the purpose of the qualitative comparison between the two models is to demonstrate the effect of shell on the MB dynamics. The behavior of the bubble in the absence of shell is used as a reference to reveal the effect of the enhanced non-linearities due to coating at low excitation pressures. The Marmottant model [62] is written a popular form [59] and addresses the inaccuracies of the Keller-Miksis model when $\frac{\left|\overset{˙}{R}\right|}{c}\approx 1$. We show in Appendix A, that in the absence of the shell terms in the Marmottant model the radial oscillations of the bubbles as predicted by the two models are relatively in good agreement. Radial oscillation amplitude of the periodic behavior, the pressure threshold of the onset of various nonlinear regimes and the chaotic behavior are in good agreement. However, when the oscillations are chaotic the radial oscillation amplitudes as predicted by both models are not always equal.

### 2.3 Investigation tools

Bifurcation diagrams are valuable tools to analyze the dynamics of nonlinear systems since qualitative and quantitative changes of the dynamics of the system can be investigated effectively over a wide range of control parameters. In this paper, we employ a more comprehensive bifurcation analysis method introduced in [73], [74].

#### 2.3.1 Conventional bifurcation analysis (Poincaré cross section at each driving period)

When dealing with systems responding to a driving force, to generate the points in the bifurcation diagrams vs. the control parameter, one option is to sample the R(t) curves using a specific point in each driving period. The approach can be summarized in:

(4) $$P\equiv (R(\Theta ))\{(R(t),\overset{˙}{R}(t)):\Theta =\frac{n}{f}\}\mathrm{wheren}=100,101\ldots 150$$

where *P* denotes the points in the bifurcation diagram, *R* and $\overset{˙}{R}$ are the time dependent radius and wall velocity of the bubble at a given set of control parameters of ($R_{0},P_{0},P_{A},c,k,\mu$, σ,f) and Θ is given by $\frac{n}{f}$. Points on the bifurcation diagram are constructed by plotting the solution of R(t) at time points that are multiples of the driving acoustic period. In this work, the results are plotted for n=100-150 to ensure a steady state solution has been reached.

#### 2.3.2 Method of maxima

As a more general method, bifurcation points can be constructed by setting one of the phase space coordinates to zero:

(5) $$Q\equiv \max(R)\{(R,\overset{˙}{R}):\overset{˙}{R}=0\}$$

In this method, the steady state solution of the radial oscillations for each control parameter is considered. The maxima of the radial peaks ($\overset{˙}{R}=0$) are identified (determined within 100–150 cycles of the stable oscillations) and are plotted versus the given control parameter in the bifurcation diagrams. The bifurcation diagrams of the normalized bubble oscillations ($\frac{R}{R_{0}}$) are calculated using both methods a) and b). When the two results are plotted alongside each other, it is easier to uncover more important details about the SuH and UH oscillations, as well as the SH and chaotic oscillations.

## 3 Results

### 3.1 Resonance curves

Compared to uncoated bubbles and coated bubbles with pure viscoelsatic behavior (e.g. Keller-Miksis model [81], Hoff model [82], Morgan model [83]), the resonance behavior of lipid coated bubbles are more complex. This is due to the buckling and rupture of the shell and dynamic variation of the effective surface tension of the bubble. As an example [68], [77] have shown numerically and experimentally that a pressure increase leads to a significant displacement of the main resonance (frequency of maximum response) of the bubble leading to a significant shift of the resonance curve.

**Fig. 2:**
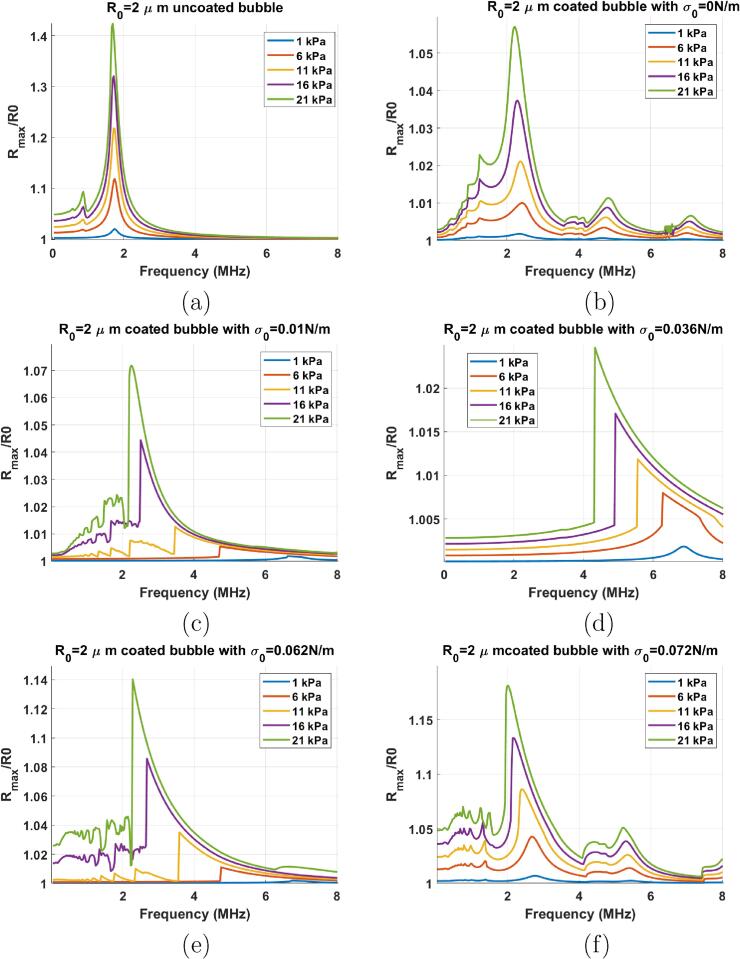
Resonance curves of a bubble with $R_{0}=2\mu m$ at different pressures for: a) uncoated bubble, and the coated bubble with b) $\sigma_{0}=0\;N/m$, c) $\sigma_{0}=0.01\;N/m$, d) $\sigma_{0}=0.036\;N/m$, e) $\sigma_{0}=0.062\;N/m$ & f) $\sigma_{0}=0.072\;N/m$.

Fig. 2 compares the resonance curves of a 2μm bubble at excitation pressure amplitudes 1, 6, 11, 16 & 21 kPa. In order to better understand the effect of the initial surface tension we have presented the case of the uncoated bubble in Fig. 2a & the coated bubbles with $\sigma_{0}$ of 0, 0.01, 0.036, 0.062 & 0.072 N/m in Fig. 2b–f respectively. The shell parameters for the bubble model are χ=3.5N/m & $k_{s}=4\;*\;10^{-9}\;\mathrm{kg}/s$ [80], [84].

Upon a first glance at Fig. 2, the high sensitivity of the coated bubble to $\sigma_{0}$ is evident. While the resonance frequency of the uncoated bubble decreases slightly from ≈1.77 MHz to ≈1.69 MHz, the resonance frequency of the lipid coated bubble changes considerably over this relatively small pressure amplitude range (1 kPa–21 kPa). The resonance frequency ($f_{r}$) change as a function of $P_{A}$ significantly depends on the $\sigma_{0}$. The bubbles with $\sigma_{0}=0.01$ & 0.062 N/m display the largest change in $f_{r}$ (fundamental frequency of the maximum response) which manifests itself in a skewness [68] in the resonance curve (Fig. 2c & e). Meanwhile, the coated bubbles with $\sigma_{0}=0$ N/m (at buckling stage) & $\sigma_{0}=0.072$ N/m (at rupture state) display the least change in the resonance frequency; however, in both cases 1/2 and 1/3 subharmonic (SH) resonances are generated at the lowest pressure thresholds. The reason for large change in the $f_{r}$ of the bubble with $\sigma_{0}=0.01$ & 0.062 N/m is that $R_{0}$ is very close to $R_{b}$ and $R_{r}$ respectively, thus these bubbles are most sensitive to variations in effective surface tension as the pressure amplitude increases.

**Fig. 3:**
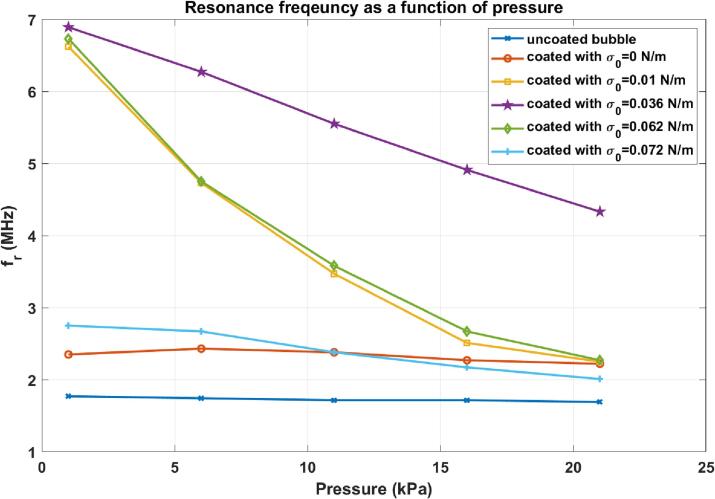
Resonance frequency as a function of pressure amplitude for the bubbles in Fig. 1.

**Fig. 4:**
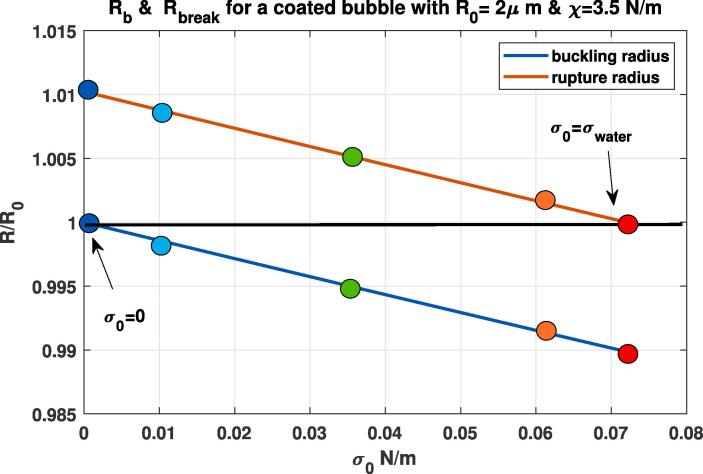
$R_{b}$ (blue curve) and $R_{\mathrm{rupture}}$ (red curve) as a function of $\sigma_{0}$. The circles mark the $R_{b}$ & $R_{\mathrm{rupture}}$ with ones in blue corresponding to $\sigma_{0}=0\;N/m$, light blue corresponding to $\sigma_{0}=0.01\;N/m$, green corresponding to $\sigma_{0}=0.036\;N/m$, orange corressponding to $\sigma_{0}=0.062\;N/m$ & red circles corresponding to $\sigma_{0}=0.072\;N/m$. (For interpretation of the references to colour in this figure legend, the reader is referred to the web version of this article.)

The resonance frequency ($f_{r}$) as function of pressure amplitude is shown in Fig. 3. At 1 kPa, the bubble with $\sigma_{0}=0.035$ N/m has the highest resonance frequency. A pressure amplitude increase to 11 kPa results in a large change in the $f_{r}$ of the bubbles with $\sigma_{0}=0.01$ N/m (6.62 to 3.47 MHz), $\sigma_{0}=0.062$ N/m (from 6.73 to 3.58 MHz) & for $\sigma_{0}=0.036$ N/m (6.89 MHz to 5.55 MHz). The uncoated bubble, and the bubbles with $\sigma_{0}=0$ & $\sigma_{0}=0.072$ N/m display very small changes in the $f_{r}$ as pressure amplitude increases from 1 kPa to 5 kPa. The bubbles with $\sigma_{0}=0$ N/m and $\sigma_{0}=0.072$ N/m have the largest $R_{r}$ and $R_{b}$ respectively and a larger pressure is needed to change the state of the coating from buckled to rupture and vice versa. Fig. 4 displays the buckling and the rupture radii as a function of $\sigma_{0}$. The bubble with $\sigma_{0}=0$ N/m is initially at the buckled state, and has the largest rupture radius of $\approx 1.0102R_{0}$. The bubble with $\sigma_{0}=0.072$ N/m is initially at the ruptured state and has the lowest buckling radius of $\approx 0.989R_{0}$. Thus, for these two bubbles higher acoustic pressures are required to change the state of the coating and consequently the rates of change of their main resonance ($f_{r}$) with pressure are the smallest. The bubble with $\sigma_{0}=0.01$ N/m buckles at $R_{b}=0.9986R_{0}$ and the bubble with $\sigma_{0}=0.062$ N/m ruptures at $\approx 1.0014R_{0}$, thus a very small pressure excitation is able to change the state of the coating to buckled or ruptured respectively. Hence, these two bubbles display the highest rates of change of $f_{r}$ with increasing pressure. The relationship between $R_{b},R_{r}$ and χ and $\sigma_{\mathrm{rupture}}$ are further explored in Appendix B.

Similar to our previous work in [34], [37], [38], [56], in this work we will attempt to classify the nonlinear dynamics of the lipid bubbles as a function of pressure amplitude when they are sonicated with fractions or multiples of their $f_{r}$. However, the initial sharp decrease of the resonance frequency with pressure will make the classification difficult. Moreover, characterization of the coating parameters of the bubbles in experiments are generally through attenuation measurements of the bubble solution when there is an excitation pressure amplitude above 1 kPa is applied. As an instance negative peak pressure amplitude of 25 kPa, 12.5 kPa, 30 kPa, 10 kPa & 5 kPa were applied respectively in [85], [86], [87], [80], [84] and peak to peak pressures of 33 kPa were applied in [87]. Very low pressures can not be applied experimentally due to the signal to noise constraints of the measurements systems.

To simplify the classification method and to have a better comparison with published experimental data we have calculated the resonance frequency at $P_{a}=10\;\mathrm{kPa}$ and used it for further study. Thus, in this paper for coated bubbles $f_{r}$ refers to the resonance frequency at $P_{a}=10\;\mathrm{kPa}$.

### 3.2 Radial oscillations as a function of time and the corresponding changes in the σ(R)

**Fig. 5:**
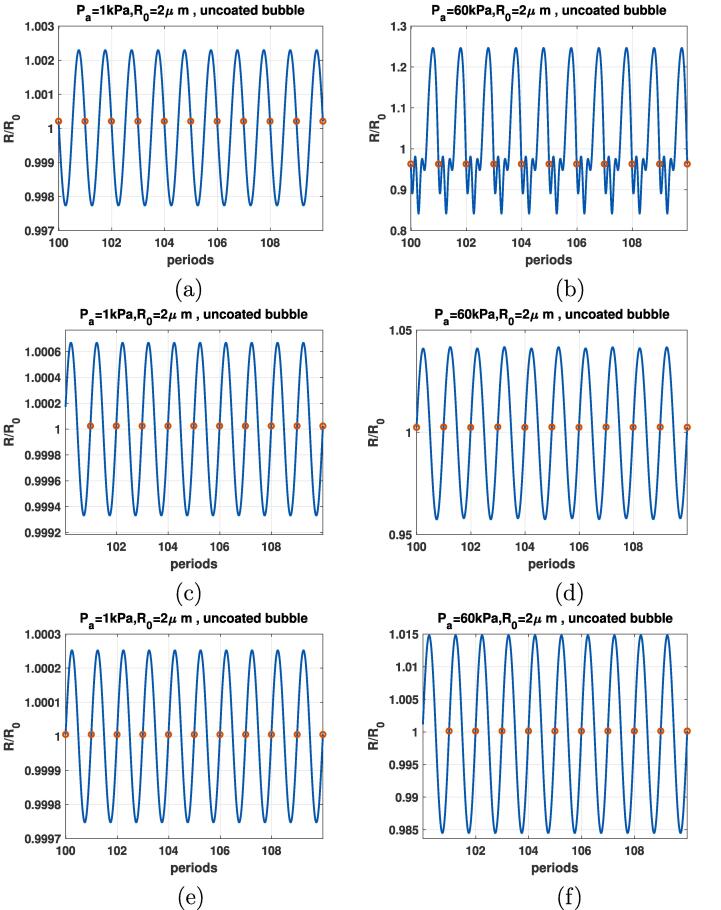
$R/R_{0}$ as function of the driving periods for a C3F8 uncoated bubble with $R_{0}=2\;\mu m$ when: a) $f=0.3f_{r}$ & $P_{a}=1\;\mathrm{kPa}$, b) $f=0.3f_{r}$ & $P_{a}=60\;\mathrm{kPa}$, c) $f=2f_{r}$ & $P_{a}=1\;\mathrm{kPa}$, d) $f=2f_{r}$ & $P_{a}=60\;\mathrm{kPa}$, e) $f=3f_{r}$ & $P_{a}=1\;\mathrm{kPa}$ & f) $f=3f_{r}$ & $P_{a}=60\;\mathrm{kPa}$. Red circles correspond to the location of R(t) at each period.

In Fig. 2, we observed the generation of SuH as well as SH resonances at very low pressures in case of the coated bubbles. In this section, the enhanced nonlinear oscillations and their relationship with the bubble surface tension are briefly investigated to have a better insight on the mechanisms of enhanced nonlinearity. Fig. 5 shows the radial oscillations of the uncoated bubble as a function of 10 acoustic driving periods (100–110). The left column shows the radial oscillations when $P_{a}=1\;\mathrm{kPa}$ and $f=0.3f_{r},2f_{r}$ and $3f_{r}$ in Fig. 5a, c and e respectively. right column shows the radial oscillations when $P_{a}=60\;\mathrm{kPa}$ and $f=0.3f_{r},2f_{r}$ and $3f_{r}$ in Fig. 5b, d and f respectively. The red circles locate the amplitude of the radial oscillations at each period. This is the Poincaré cross section at each driving period which is used to generate the bifurcation diagram using the method introduced in 2.3.1. The bubble oscillations in Fig. 5 are period 1 (P1) and the red circles have the same value at each driving periods. This indicates the absence of any SHs. Only 3rd order SuHs are seen (P1 oscillations with 3 maxima) when pressure amplitude is 60 kPa in Fig. 5b.

**Fig. 6:**
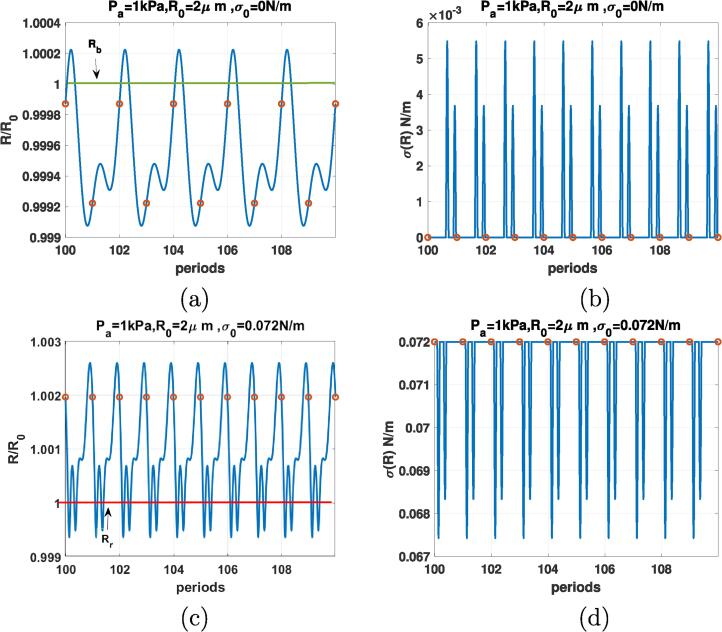
$R/R_{0}$ (left) & σ(R) (right) as function of the driving periods for a C3F8 coated bubble with $R_{0}=2\;\mu m$ when $f=0.3f_{r}$ & $P_{a}=1\;\mathrm{kPa}$ for: a & b-$\sigma_{0}=0\;N/m$, c& d-$\sigma_{0}=0.072\;N/m$. Red circles correspond to the location of R(t) at each period. The green and red horizontal lines mark the buckling and rupture radii respectively. (For interpretation of the references to colour in this figure legend, the reader is referred to the web version of this article.)

Fig. 6, depicts the case of the coated bubble with $R_{0}=2\mu m$ when $f=0.3f_{r}$ and $P_{a}=1\;\mathrm{kPa}$. Top row is for $\sigma_{0}=0$ N/m with radial oscillations in Fig. 6a and the corresponding σ(R) in Fig. 6b. The oscillations are P1 (red circle only represents one value), however, the radial oscillations have two maxima, indicating a 2nd order SuH regime of oscillations. The corresponding σ(R) drops to zero and stays zero in the buckled state until the bubble expands above the buckling radius and again drops to zero when the bubble buckles upon compression. The bubble with σ(R)=0.072 N/m (Fig. 6c) exhibits P1 oscillation with 3 maxima and thus a 3rd order SuH regime. When the bubble expands, σ(R) can not grow beyond the surface tension of water (0.072 N/m) thus the σ(R) curve becomes flat(Fig. 6d). Upon contraction σ(R) decreases and upon expansion it grows until the coating breaks and surface tension becomes equal to 0.072 N/m. In both cases, the buckling and rupture of the shell results in the enhanced nonlinearity (in these cases enhanced SuHs). For the bubble with $\sigma_{0}=0$ N/m there is compression dominated behavior and for the bubble with $\sigma_{0}=0.072$ N/m expansion dominated behavior is observed.

**Fig. 7:**
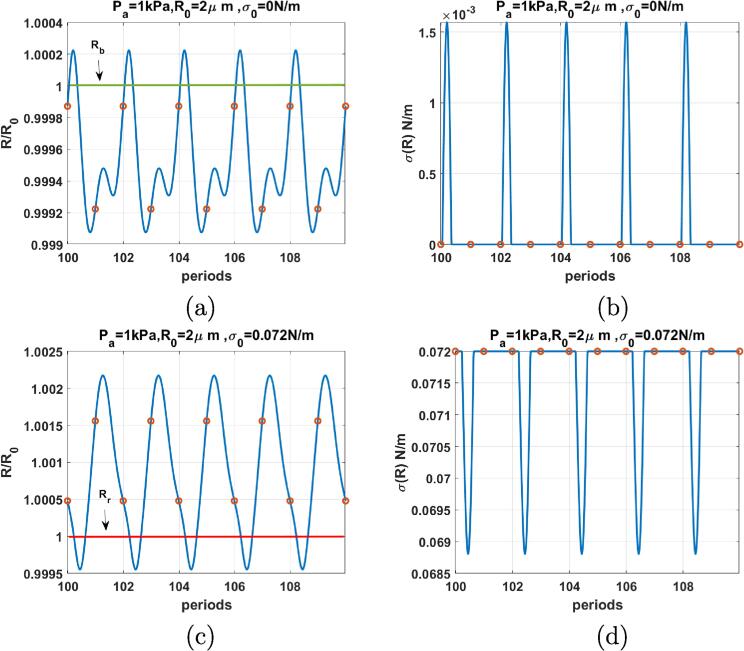
$R/R_{0}$ (left) & σ(R) (right) as function of the driving periods for a C3F8 coated bubble with $R_{0}=2\;\mu m$ when $f=2f_{r}$ & $P_{a}=1\;\mathrm{kPa}$ for: a & b-$\sigma_{0}=0\;N/m$, c& d-$\sigma_{0}=0.072\;N/m$. Red circles correspond to the location of R(t) at each period. The green and red horizontal lines mark the buckling and rupture radii respectively. (For interpretation of the references to colour in this figure legend, the reader is referred to the web version of this article.)

Fig. 7, depicts the case of the coated bubble with $R_{0}=2\mu m$ when $f=2f_{r}$ and $P_{a}=1\;\mathrm{kPa}$. For $\sigma_{0}=0$ N/m (Fig. 7a) compression dominated radial oscillations are P2 (red circle corresponds to two values). The corresponding σ(R) (Fig. 7b) remains equal to zero for a time duration of less than two periods followed by a short spike when the bubble expands above the buckling radius. The surface tension exhibits 5 spikes for the duration of 10 cycles. The bubble with σ(R)=0.072 N/m (Fig. 7c) exhibits expansion dominated P2 oscillation with 1 maximum. The σ(R) curve (Fig. 7d) exhibits the same behavior of Fig. 7b with an inverted shape. The surface tension displays 5 inverted spikes within 10 cycles.

**Fig. 8:**
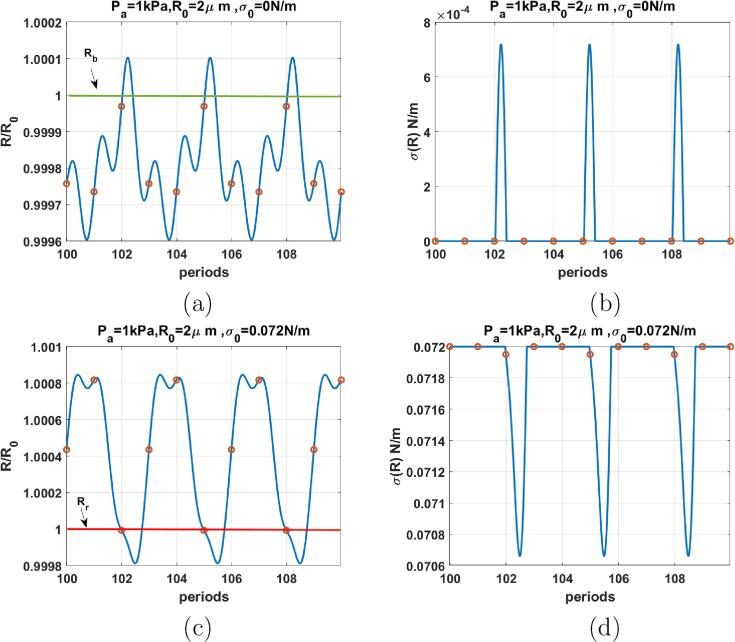
$R/R_{0}$ (left) & σ(R) (right) as function of the driving periods for a C3F8 coated bubble with $R_{0}=2\;\mu m$ when $f=3f_{r}$ & $P_{a}=1\;\mathrm{kPa}$ for: a & b-$\sigma_{0}=0\;N/m$, c& d-$\sigma_{0}=0.072\;N/m$. Red circles correspond to the location of R(t) at each period. The green and red horizontal lines mark the buckling and rupture radii respectively. (For interpretation of the references to colour in this figure legend, the reader is referred to the web version of this article.)

Fig. 8, depicts the case of the coated bubble with $R_{0}=2\mu m$ when $f=3f_{r}$ and $P_{a}=1\;\mathrm{kPa}$. For $\sigma_{0}=0$ N/m (Fig. 8a) compression dominated radial oscillations are P3 (red circle corresponds to three values) with 3 maxima. The corresponding σ(R) (Fig. 8b) remains zero for a time duration of less than three periods followed by a short spike when the bubble expands above buckling radius. The surface tension exhibits 3 spikes for the duration of 10 cycles. The bubble with σ(R)=0.072 N/m (Fig. 8c) exhibits expansion dominated P3 oscillation with 2 maxima. The σ(R) curve (Fig. 8d) exhibits the same behavior of Fig. 8b with an inverted shape. The surface tension displays 3 inverted spikes within 10 cycles.

**Fig. 9:**
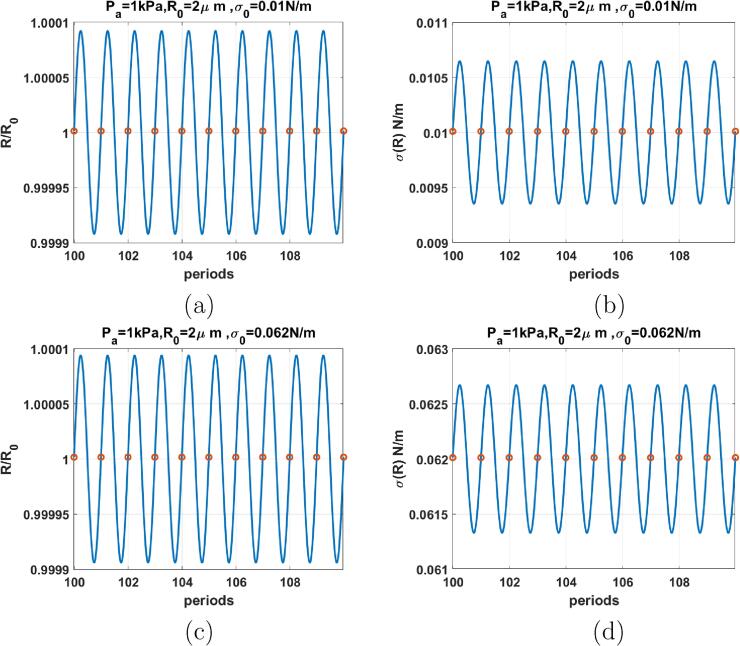
$R/R_{0}$ (left) & σ(R) (right) as function of the driving periods for a C3F8 coated bubble with $R_{0}=2\;\mu m$ when $f=3f_{r}$ & $P_{a}=1\;\mathrm{kPa}$ for: a & b-$\sigma_{0}=0.01\;N/m$, c& d-$\sigma_{0}=0.062\;N/m$. Red circles correspond to the location of R(t) at each period. The green and red horizontal lines mark the buckling and rupture radii respectively. (For interpretation of the references to colour in this figure legend, the reader is referred to the web version of this article.)

**Fig. 10:**
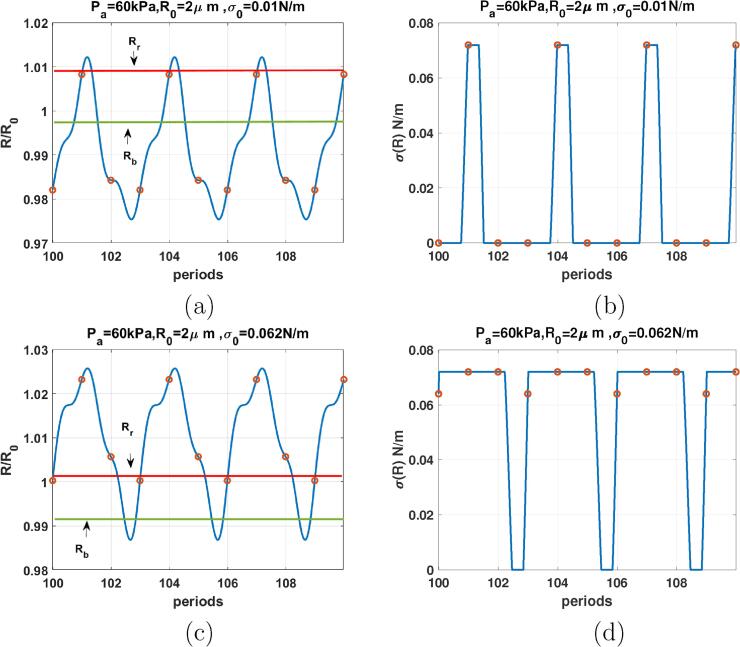
$R/R_{0}$ (left) & σ(R) (right) as function of the driving periods for a C3F8 coated bubble with $R_{0}=2\;\mu m$ when $f=2f_{r}$ & $P_{a}=60\;\mathrm{kPa}$ for: a & b-$\sigma_{0}=0.01\;N/m$, c& d-$\sigma_{0}=0.062\;N/m$. Red circles correspond to the location of R(t) at each period. The green and red horizontal lines mark the buckling and rupture radii respectively. (For interpretation of the references to colour in this figure legend, the reader is referred to the web version of this article.)

Fig. 9 shows the radial oscillations and the surface tension of the coated bubble with $R_{0}=2\;\mu m$ at $P_{a}=1\;\mathrm{kPa}$ as a function of periods for bubbles with $\sigma_{0}=0.01$ N/m (Fig. 9a–b) and $\sigma_{0}=0.062$ N/m (Fig. 9c–d). Both cases display a P1 oscillations with symmetric amplitude around the initial bubble radius. The σ(R) curves display symmetric oscillations and absence of sharp spikes that are seen in Fig. 6, Fig. 7, Fig. 8. When $P_{a}$ increases the coating can buckle or rupture. Fig. 10 shows the radial oscillations and surface tension of the coated bubble with $R_{0}=2\;\mu m$ at $P_{a}=60\;\mathrm{kPa}$ as a function of periods for bubbles with $\sigma_{0}=0.01$ N/m (Fig. 10a–b) and $\sigma_{0}=0.062$ N/m (Fig. 10c–d). Both cases display P3 oscillations and 3 spikes in the σ(R) within 10 periods.

Comparison between Fig. 5, Fig. 6, Fig. 7, Fig. 8, Fig. 9, Fig. 10 shows that the sharp variations of the σ(R) in the neighborhood of the buckling or rupture radii enhances the nonlinear behavior. The coated bubbles initially at buckled or ruptured state display this behavior at a pressure amplitude as low as 1 kPa. The coated bubbles with $\sigma_{0}=0.01$ N/m and $\sigma_{0}=0.062$ N/m need slightly higher pressures for the enhanced nonlinear oscillations. The uncoated bubble did not show any enhanced nonlinearity.

### 3.3 Bifurcation structure of the uncoated bubble

In this section, we briefly highlight the main nonlinear regimes of the dynamics of the uncoated bubble as a function of pressure amplitude at different frequencies. This data will be useful when analyzing the behavior of the lipid coated bubble by highlighting the shell effects on the coated bubble dynamics. The red curve is constructed using the method of maxima (Section 3) and the blue curve is constructed using the Poincaré cross section at each driving period.

**Fig. 11:**
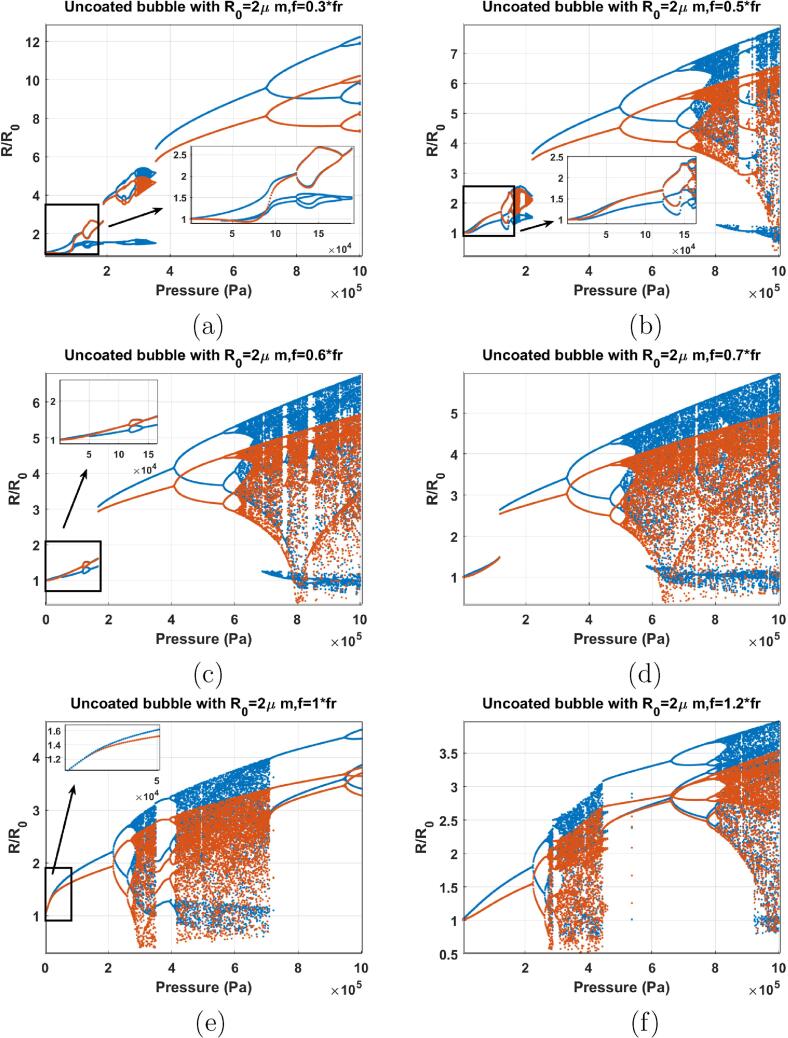
Bifurcation structure of the $R/R_{0}$ of the C3F8 uncoated bubble with $R_{0}=2\;\mu m$ as a function of pressure amplitude when: a) $f=0.3f_{r}$, b) $f=0.5f_{r}$, c) $f=0.6f_{r}$, d) $f=0.7f_{r}$, e) $f=f_{r}$ & f) $f=1.2f_{r}$.

Fig. 11a shows the bifurcation structure of the uncoated bubble with $R_{0}=2\mu m$ sonicated with $f=0.3f_{r}$. Pressure increase above ≈50kPa leads to the generation of 3 maxima in the bubble oscillations (3 blue lines) for a period 1 (P1) oscillation regime. Thus 3rd order SuH regime [57] is generated. In the regime of 3rd order SuH oscillations, the frequency component at 3f is stronger than the rest of the frequency components of the scattered pressure. Oscillations undergo period doubling (PD) at about 124 kPa. The blue curve with 3 maxima undergoes PD concomitant with the 1 PD in the red curve; thus oscillations become P2 with 6 maxima and 7/2 order UH oscillations are generated ($124\mathrm{kPa}<P_{a}<178\;\mathrm{kPa}$). When 7/2 order UHs occur the frequency component at 3.5f in the scattered pressure by the bubble is stronger than the frequency components at 0.5f, 1.5f, 4.5f, etc. The 3rd order region and the 7/2 order UH region are highlighted as an inset in Fig. 11a. Further pressure increase leads to SN bifurcation to 2nd order SuH oscillations of higher amplitude, followed by 5/2 UHs, and a small chaotic window. Finally a giant P1 resonance emerges out of the chaotic window undergoing further PDs at higher pressures.

When $f=0.5f_{r}$ (Fig. 11b), as pressure amplitude increases above 14 kPa, 2 maxima are generated in the P1 oscillation regime (2nd order SuH). Further pressure increase results in a PD in both the blue and red graphs leading to a P2 oscillation with 4 maxima (5/2 UH oscillations). This region is highlighted as an inset in Fig. 11b. Chaos occurs in a small window above 160 kPa with a tiny window of periodic (P3 with 5 maxima) behavior within. Afterwards, a giant P1 resonance emerges out of the chaotic window. The P1 oscillations undergo a multiple cascades of PDs to chaos.

When $f=0.6f_{r}$ (Fig. 11c) 5/2 UH oscillations (P2 with 4 maxima) are developed and then transition to P1 oscillations through a bubble in the pressure window of 116–150 kPa (highlighted in an inset). P1 oscillations then undergo a saddle node bifurcation to a P1 oscillation with higher amplitude at $P_{a}\approx 166\;\mathrm{kPa}$. This is due to the pressure dependent resonance behavior that has been discussed in detail in [56]. Further pressure increase leads to a PD to P2 oscillations (at 406 kPa) which is followed by a cascade of PDs to chaos at ≈614kPa.

The dynamics of the bubble sonicated with $f=0.7f_{r}$ (Fig. 11d) is similar to the case of $f=0.6f_{r}$; however, 5/2 UH oscillations are not generated and SN bifurcation occurs at a slightly lower pressure amplitude (117 kPa). At this pressure amplitude the red curve meets the blue curve. This is the pressure dependent resonance and the wall velocity becomes in phase with the driving signal. This is discussed in detail with numerical and experimental observations in [86]. PD occurs at 326 kPa which is lower than the PD threshold in Fig. 11c. Chaos settles through a cascade of PDs at 504 kPa.

When $f=f_{r}$ (Fig. 11e) oscillations are P1 and the blue line and the red line have the same value (highlighted in the inset) which indicates that the wall velocity is in phase with the acoustic driving force due to the resonance (page 290 in [92]). The two curves start diverging as soon as pressure increases above 18 kPa and at 215 kPa the oscillations undergo PD. Oscillations become chaotic above 400 kPa with a small window of periodic behavior (P3 with 3 maxima).

When $f=1.2f_{r}$ (Fig. 11f), we witness the similar behavior as the case of $f=f_{r}$; however, P2 oscillations are developed for $R_{\max}/R_{0}<2$, thus P2 oscillations are more likely stable [93].

**Fig. 12:**
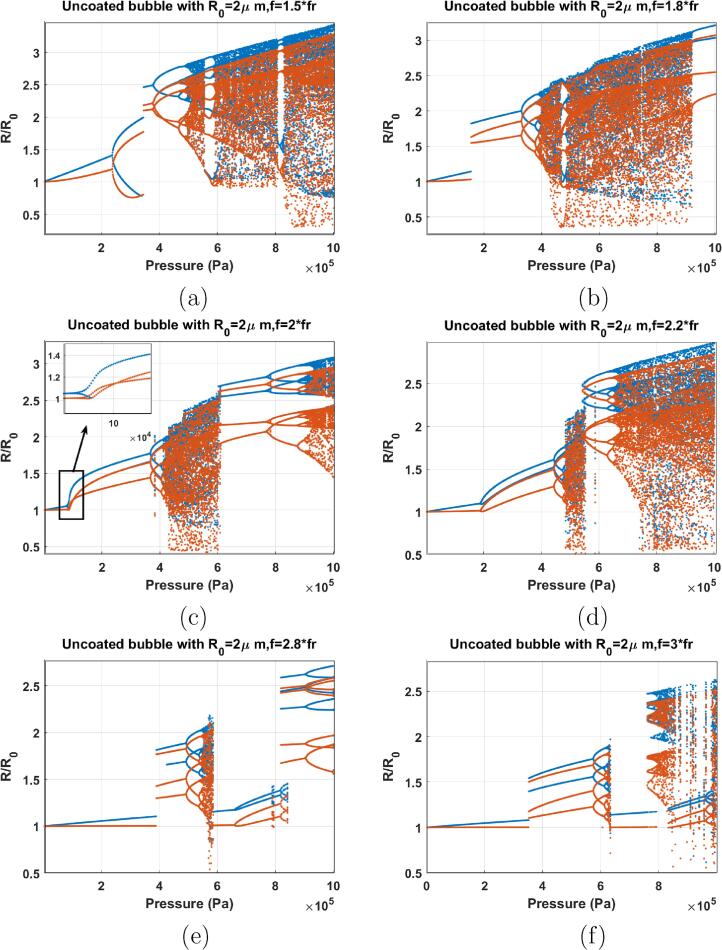
Bifurcation structure of the $R/R_{0}$ of the C3F8 uncoated bubble with $R_{0}=2\;\mu m$ as a function of pressure amplitude when: a) $f=1.5f_{r}$, b) $f=1.8f_{r}$, c) $f=2f_{r}$, d) $f=2.2f_{r}$, e) $f=2.8f_{r}$ & f) $f=3f_{r}$.

When $f=1.5f_{r}$ (Fig. 12a), P1 oscillations undergo PD with 2 maxima at 236 kPa. P2 oscillations undergo a SN bifurcation to P2 oscillations of higher amplitude at 347 kPa. The SN bifurcation is coincident with the pressure dependent SH resonance (${\mathrm{Pdf}}_{\mathrm{sh}}$) [58]. This results in the over-saturation and enhancement of the SH signal from the pressure scattered by bubbles [58]. P2 oscillations undergo successive PDs to chaos at ≈494kPa.

When $f=1.8f_{r}$ (Fig. 12b) P1 oscillations undergo a SN bifurcation to P2 oscillations of higher amplitude at 155 kPa. The P2 oscillations amplitude $\frac{R_{\max}}{R_{0}}<2$ thus bubbles may have higher stability compared to Fig. 12a. Further pressure amplitude increase leads to chaos through successive PDs. At 931 kPa a giant P3 resonance emerges out of the chaotic window.

When $f=2f_{r}$ (linear SH resonance frequency), PD occurs at the lowest pressure threshold of 77 kPa (highlighted in an inset) [34]. P2 oscillations undergo successive PDs and chaos appears at 400 kPa and extends to ≈600kPa where giant P3 resonance emerges out of the chaotic window. Oscillations later become chaotic again through successive PDs.

When $f=2.2f_{r}$ (Fig. 12d), PD occurs at 189 kPa which is higher than the PD threshold when $f=2f_{r}$. P2 oscillations undergo PD to P4-2 at 445 kPa and then are followed by chaos through consecutive PDs at 482 kPa.

The case of $f=2.8f_{r}$ is depicted in Fig. 12e. P1 oscillations undergo a SN to P3 oscillations at 390 kPa. P3 oscillations undergo PD to P6 at 489 kPa and a small chaotic window appears at 587 kPa. Chaos disappears and low amplitude P1 emerges out of the chaotic window at 588 kPa which later undergo a PD similar to Fig. 12d at 661 kPa. Further pressure increase results in the occurrence of P4 through a SN at 819 kPa. P4 oscillations undergo PD to P8 at about 900 kPa.

When $f=3f_{r}$ (Fig. 12f), P3 occurs at 353 kPa through SN bifurcation. P3 extends to 567 kPa where P6 oscillations are generated through a PD. A small chaotic window appears before the low amplitude P1 which then undergoes a SN to P8 oscillations. Finally chaos is generated at ≈800kPa.

### 3.4 Bifurcation structure of the coated bubble with $\sigma_{0}=0$ & $\sigma_{0}=0.072$ N/m

Due to the sharp decrease of resonance frequency with pressure amplitude and for simplification of the comparisons, as well as to consider the experimental constrains $f_{r}$ is chosen to be the frequency of maximum response at 10 kPa. For the bubble with $\sigma_{0}=0N/m,f_{r}=f_{10\mathrm{kPa}}≊f_{1\mathrm{kPa}}$. For the bubble with $\sigma_{0}=0.072N/m,f_{r}=f_{10\mathrm{kPa}}=0.865f_{1\mathrm{kPa}}$.

**Fig. 13:**
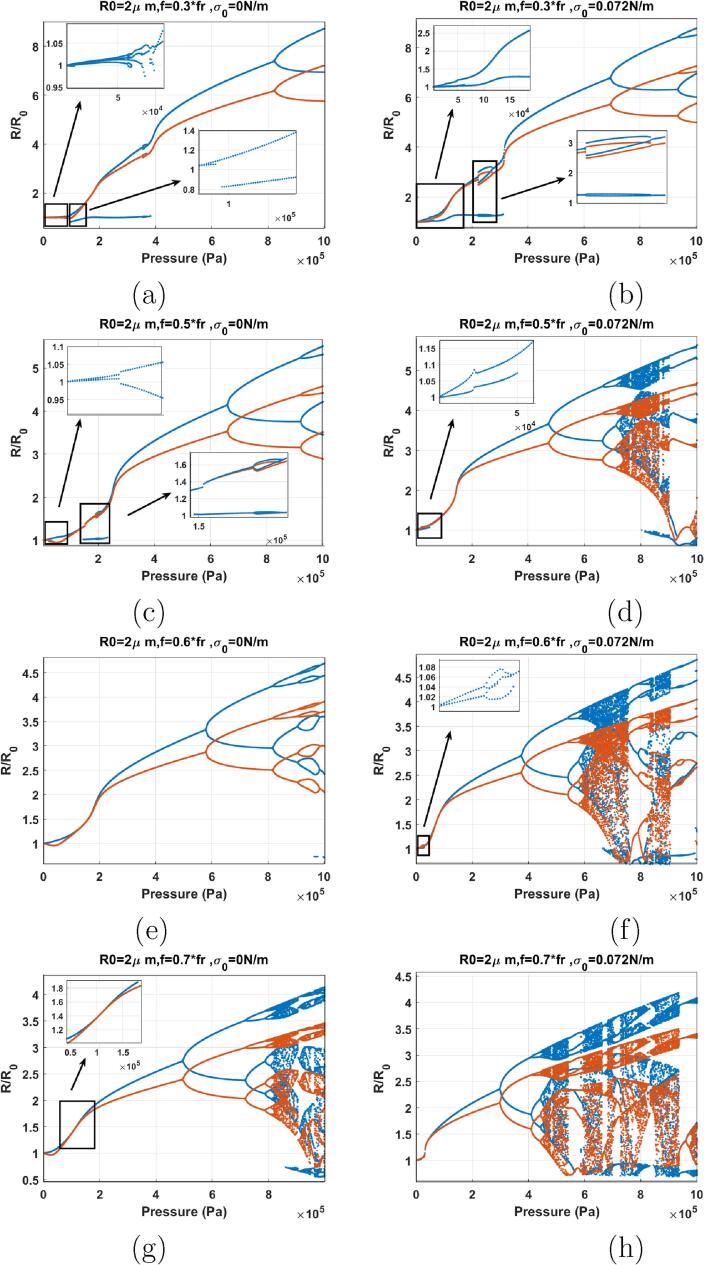
Bifurcation structure of the $R/R_{0}$ of the C3F8 coated bubble with $R_{0}=2\;\mu m$ as a function of pressure amplitude (left: $\sigma_{0}=0\;N/m$ and right: $\sigma_{0}=0.072\;N/m$): a–b) $f=0.3f_{r}$, c–d) $f=0.5f_{r}$, e–f) $f=0.6f_{r}$, g–h) $f=0.7f_{r}$.

Fig. 13a–b show the bifurcation structure of the coated bubble when $f=0.3f_{r}$ & $\sigma_{0}=0$ (a) and $\sigma_{0}=0.072$ (b). This assumes the coatings are initially in the buckled and ruptured states receptively. The bubbles start oscillation in a P1 with two maxima (2nd order SuH) right from $P_{a}=1\;\mathrm{kPa}$. The following evolution 2nd order SuH → 3rd order SuH (P1 with 3 maxima) → 4th order SuH (P1 with 4 maxima) takes place as pressure amplitude increases (these are highlighted as insets in Fig. 13a–b). Compared to the uncoated bubble case, the 2nd order SuH appears at a very small pressure threshold ($P_{a}=1\;\mathrm{kPa}$). Wall velocity is in phase with the driving acoustic pressure for most of the pressures below 200 kPa. Further pressure amplitude increases results in the gradual disappearance of the maxima, and above 210 kPa, only two maxima remain in the bubble oscillations for both cases. The radial oscillation amplitude increases, until PD occurs in both graphs and 5/2 UH resonance occur (P2 oscillations with 4 maxima which is highlighted as an inset in Fig. 13b). For $\sigma_{0}=0$ N/m, 5/2 UH resonance exists for $P_{a}=431-450\;\mathrm{kPa}$ & for $\sigma_{0}=0.072$ N/m, 5/2 UH resonance exist for $P_{a}=330-365\;\mathrm{kPa}$. The UH resonance occurs and disappears through a bubbling bifurcation. 2nd maxima is annihilated soon after the disappearance of UH. Further pressure amplitude increase results in PD at very large oscillation amplitudes $\frac{R_{\max}}{R_{0}}>5$ where the bubble may not sustain non-destructive oscillations.

When $f=0.5f_{r}$ (Fig. 13c–d), oscillations start with 2nd order SuH oscillations (P1 with 2 maxima) right from the start at 1 kPa and this stretches to ≈50kPa in both cases at which point 2nd maxima disappears (highlighted as insets in Fig. 13c–d). For the case of $\sigma_{0}=0$ N/m (Fig. 13c), second maxima re-appear at 147 kPa. At 190–231 kPa a bubbling bifurcation occurs where the oscillations become P2 with 4 maxima (5/2 UH regime which is highlighted as an inset). The second maxima disappears again at 230 kPa. Wall velocity stay in phase for most of the driving acoustic pressure range of $P_{a}<262\;\mathrm{kPa}$ for $\sigma_{0}=0$ N/m & $P_{a}<151\;\mathrm{kPa}$ for $\sigma_{0}=0.072$ N/m. Further pressure increase results in PD ($P_{a}=660\;\mathrm{kPa}$ for $\sigma_{0}=0$ N/m & $P_{a}=473\;\mathrm{kPa}$ for $\sigma_{0}=0.072$ N/m).

Compared to the uncoated bubble case, the coating at its ruptured or buckled state reduces the pressure threshold for SuH oscillations. UH oscillations, however, are suppressed and only occur at higher pressures and for a much shorter range of excitation pressures. The pressure threshold for the giant PD increases and chaotic oscillations are suppressed within the excitation pressure amplitude range that is examined here. This can be due to the increased damping due to the coating.

When $f=0.6f_{r}$ (Fig. 13e–f), oscillations are P1 and above a pressure threshold (100 kPa for $\sigma_{0}=0$ N/m & 40 kPa for $\sigma_{0}=0.072$ N/m), the rate of the growth of the oscillations amplitude with pressure amplitude increases abruptly. This point is similar to a inflection point. When this occurs, the wall velocity becomes in phase with the driving acoustic pressure as the red curve has the same value of the blue curve ($100\;\mathrm{kPa}<P_{a}<189\;\mathrm{kPa}$ for $\sigma_{0}=0$ N/m & $41\;\mathrm{kPa}<P_{a}<90\;\mathrm{kPa}$ & $\sigma_{0}=0.072$ N/m). The bubble with $\sigma_{0}=0.072$ N/m undergoes a PD with 4 maxima (5/2 UHs) at ≈ 30 kPa which is highlighted as an inset in Fig. 13f. Further pressure amplitude increases results in the divergence of the blue and red curve and PD occurs at $P_{a}=576\;\mathrm{kPa}$ for $\sigma_{0}=0$ N/m & 369 kPa for $\sigma_{0}=0.072$ N/m. Oscillations undergo further PDs to P4 as pressure amplitude increases. In case of the bubble $\sigma_{0}=0$ N/m a P8 regime is created and then annihilated through a bubbling bifurcation within the P4 window. Oscillations of the bubble with $\sigma_{0}=0.072$ N/m becomes chaotic through successive PDs with intermittent windows of period behavior within.

When $f=0.7f_{r}$ (Fig. 13g–h), oscillations start in a similar manner to the case of $f=0.6f_{r}$. The growth rate of the P1 oscillation amplitude increases abruptly above a pressure threshold which is lower than the case of the $f=0.6f_{r}$ (90 kPa for $\sigma_{0}=0$ N/m & 29 kPa $\sigma_{0}=0.072$ N/m). Consequently, wall velocity becomes in phase with the driving pressure (highlighted as an inset in Fig. 13)g and further pressure amplitude increases result in the divergence of the blue and the red curve. PD occurs at $P_{a}=495\;\mathrm{kPa}$ for $\sigma_{0}=0$ N/m & 295 kPa for $\sigma_{0}=0.072$ N/m. Chaotic oscillations are finally generated through successive PDs with some periodic windows within.

The cases of the coated bubbles in Fig. 13e–h are similar to the case of the uncoated bubble sonicated with $f=0.6f_{r}$ & $f=0.7f_{r}$ (${\mathrm{Pdf}}_{r}$ [56]). However, the pressure threshold for the SN bifurcation or the increase in the growth rate of the oscillations (inflection point) is much lower in case of the coated bubble with $\sigma_{0}=0.072$ N/m despite being excited with lower frequencies. Moreover, the pressure threshold for PD and chaotic oscillations are higher for the coated bubbles with PD occurring at a higher The $\frac{R_{\max}}{R_{0}}$. This can be due to the increased damping in the bubble oscillations.

**Fig. 14:**
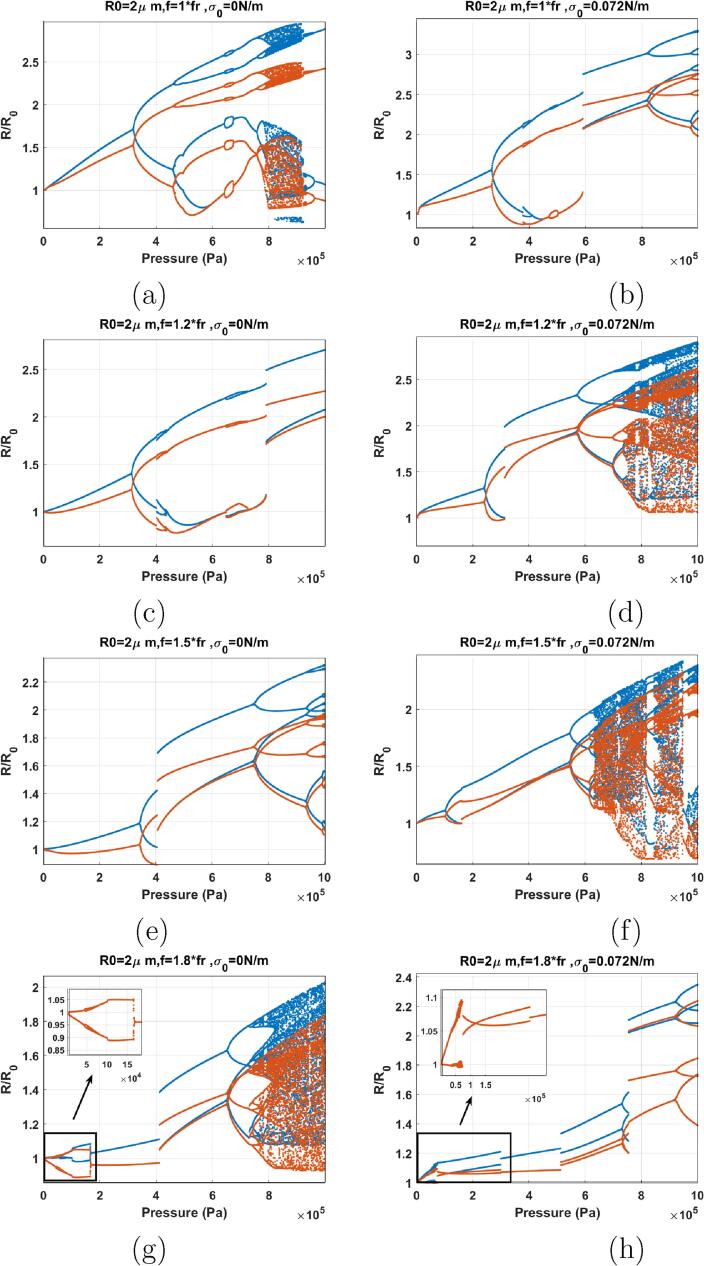
Bifurcation structure of the $R/R_{0}$ of the C3F8 coated bubble with $R_{0}=2\;\mu m$ as a function of pressure amplitude (left: $\sigma_{0}=0.0\;N/m$ and right: $\sigma_{0}=0.072\;N/m$): a–b) $f=f_{r}$, c–d) $f=1.2f_{r}$, e–f) $f=1.5f_{r}$, g–h) $f=1.8f_{r}$.

When $f=f_{r}$ (Fig. 14a-b) (note that in this paper in case of the lipid coated bubbles $f_{r}$ was considered the frequency of maximum response at 10 kPa) the red and blue curve have the same value for $P_{a}<20\;\mathrm{kPa}$. The P1 oscillations amplitude grows as pressure amplitude increases and the two curves diverge with amplitude increase. PD occurs at at $P_{a}=267\;\mathrm{kPa}$ for $\sigma_{0}=0$ N/m & 317 kPa for $\sigma_{0}=0.072$ N/m which are higher than the PD pressure amplitude for the uncoated bubble ($P_{a}=215\;\mathrm{kPa}$ (Fig. 11)e. $\frac{R_{\max}}{R_{0}}$ of the P2 oscillations of the coated bubble however, are below 2 while the oscillation amplitude of the P2 oscillations in uncoated bubble are above 2. In case of the bubble with $\sigma_{0}=0$ N/m a further pressure amplitude increase leads to P4 oscillations through another PD. P4 oscillations become P8 and then again P4 through a bubbling bifurcation; P4 oscillations later undergo a PD cascade to chaos. At $P_{a}\approx 915\;\mathrm{kPa}$ a P4 oscillation emerges out of the chaotic window through reverse PD bifurcation. P4 becomes P2 through another SB. For the bubble with $\sigma_{0}=0.072$ N/m, At $P_{a}=600\;\mathrm{kPa}$ the P2 oscillations undergo a SN bifurcation to P2 oscillations of higher amplitude. This is similar to the behavior of the uncoated bubble sonicated by its ${\mathrm{Pdf}}_{\mathrm{sh}}=\approx 1.5-1.9f_{r}$ [58] & Fig. 12a ($f=1.5f_{r}$). Thus, in case of the lipid coated bubble the buckling and rupture of the coating significantly decreases the ${\mathrm{Pdf}}_{\mathrm{sh}}$.

When $f=1.2f_{r}$ (Fig. 14c-d), the P1 oscillation amplitude increases with increasing pressure amplitude and PD occurs at $P_{a}=314\;\mathrm{kPa}$ for $\sigma_{0}=0$ N/m & 238 kPa for $\sigma_{0}=0.072$ N/m. Pressure thresholds for PD are higher than the pressure threshold of PD (218 kPa) in the uncoated bubble case in Fig. 11f. In both cases, with increasing pressure amplitude a SN bifurcation takes place from P2 to another P2 with higher amplitude ($P_{a}=796\;\mathrm{kPa}$ for $\sigma_{0}=0$ N/m & 314 kPa for $\sigma_{0}=0.072$ N/m). This is similar to the dynamics of the uncoated bubble sonicated by its ${\mathrm{Pdf}}_{\mathrm{sh}}$ [58] & Fig. 12a–b ($f=1.5f_{r}$ & $1.8f_{r}$). This shows that the dynamic variations of the effective surface tension including buckling and rupture decreases the ${\mathrm{Pdf}}_{\mathrm{sh}}$. In the case of $\sigma_{0}=0.072$ N/m chaos appears through successive PDs, however, the bubble with $\sigma_{0}=0$ N/m does not exhibit chaotic oscillations in this pressure amplitude range. Additionally at a given pressure, $\frac{R_{\max}}{R_{0}}$ is higher for the bubble with $\sigma_{0}=0.072$ N/m because of the expansion dominated behavior of the bubble. This can be one of the reasons for lower pressure threshold of P2 and chaotic oscillations in case of the bubble in ruptured state.

When $f=1.5f_{r}$ (Fig. 14e-f), the bubble behavior is similar to the uncoated bubble sonicated with its ${\mathrm{Pdf}}_{\mathrm{sh}}$. The pressure threshold for P2 oscillations are $P_{a}=338\;\mathrm{kPa}$ for $\sigma_{0}=0$ N/m & $P_{a}=98\;\mathrm{kPa}$ for $\sigma_{0}=0.072$ N/m. In case of the bubble with $\sigma_{0}=0.072$ N/m pressure threshold for PD is lower than the case of the uncoated bubble (Fig. 12a). Increasing the pressure amplitude results in a SN bifurcation from a P2 regime to a higher amplitude P2 regime. In case of the uncoated bubble the SN bifurcation results in $\frac{R_{\max}}{R_{0}}>2$, however, here P2 oscillations remain below 2 when SN occurs. The P2 oscillations undergo successive PDs to P8 in both bubbles (Fig. 14e-f). However, only the bubble with $\sigma_{0}=0.072$ N/m, exhibits chaotic oscillations. Similar to the previous cases, $\frac{R_{\max}}{R_{0}}$ is higher for the bubble in the ruptured state due to expansion dominated behavior.

When $f=1.8f_{r}$ a very interesting phenomenon is observed (Fig. 14g-h). In both cases, the bubble starts oscillating in the P2 regime at the very low pressure threshold of 1 kPa. To our best knowledge, such a low excitation threshold for P2 oscillations in nonlinear oscillators is first reported here. The dynamic of the bubble exhibits three interesting stages. The generation of P2 oscillations (at very low pressure), the disappearance of P2 oscillations and regeneration of P2 oscillations. Such behavior has been observed experimentally in [73], [94]. In [73], the disappearance of SH oscillations is referred to as an “unexpected standstill” of SHs. This will be discussed further in discussion. Within the initial P2 window, a very small P4 window occurs for both bubbles. The pressure threshold for the initiation of the P4-2 oscillations is as low as 5 kPa for the bubble with $\sigma_{0}=0.072$ N/m. The P2 oscillations disappear with increasing pressure amplitude above 173 kPa and 299 kPa for the bubbles with $\sigma_{0}=0$ & 0.072 N/m respectively. A second P2 regime re-emerges through a SN bifurcation at 412 & 514 kPa for the bubbles with $\sigma_{0}=0$ & 0.072 N/m respectively. This dynamical feature is similar to the case of uncoated bubble sonicated with its ${\mathrm{Pdf}}_{\mathrm{sh}}$ of $1.8f_{r}$ (Fig. 12b); however, the SN occurs at a higher pressure. Similar to the uncoated bubble, after the SN occurrence, the bubble with $\sigma_{0}=0$ N/m undergoes chaotic oscillations through successive PDs.

**Fig. 15:**
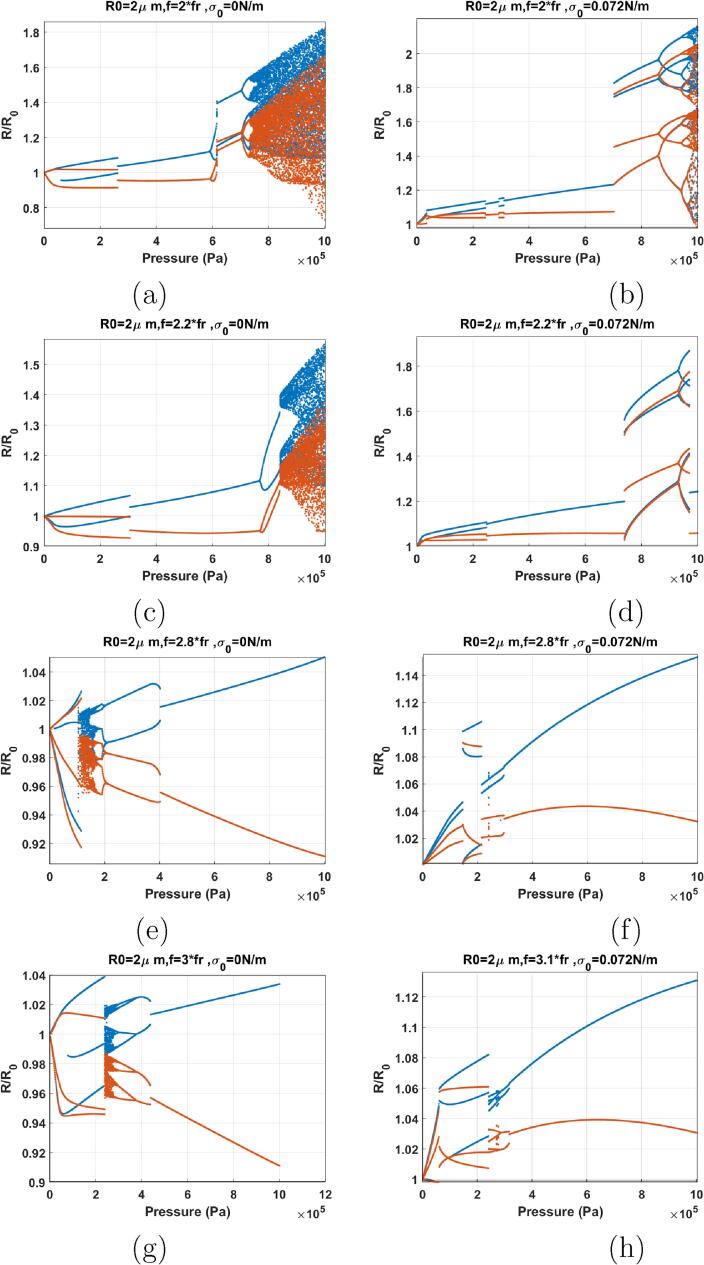
Bifurcation structure of the $R/R_{0}$ of the C3F8 coated bubble with $R_{0}=2\;\mu m$ as a function of pressure amplitude (left: $\sigma_{0}=0\;N/m$ and right: $\sigma_{0}=0.072$ N/m): a–b) $f=2f_{r}$, c–d) $f=2.2f_{r}$, e–f) $f=2.8f_{r}$, g) $f=3f_{r}$ h) $f=3.1f_{r}$.

When $f=2f_{r}$ (Fig. 15a–b), the dynamics are similar to Fig. 14g–h. P2 oscillations are generated at 1 kPa, and they disappear above 200 kPa. For the bubble with $\sigma_{0}=0$ N/m, P2 oscillations re-emerge at ≈600kPa and through a PD bifurcation. Similar to the coated bubble sonicated with its ${\mathrm{Pdf}}_{\mathrm{sh}}$ in [58], P2 oscillations undergo a SN bifurcation to P2 oscillations with higher amplitude. Further pressure amplitude increase results in chaotic oscillations through successive PDs. In case of the bubble with $\sigma_{0}=0.072$ N/m, soon after the disappearance of the P2 oscillations, a rather small window (293–310 kPa) of P2 oscillations is generated through a SN. P2 oscillations disappear and P1 oscillations undergo a SN to P3 at 707 kPa.This dynamical feature is similar to Fig. 12f where the uncoated bubble is sonicated with $f=3f_{r}$.

The dynamics of the bubble sonicated with $f=2.2f_{r}$ (Fig. 15c–d) is similar to $f=2f_{r}$ and the general dynamical features of the system stays the same.

The dynamics of the bubbles with $\sigma_{0}=0$ N/m & 0.072 N/m sonicated with $1.8f_{r}⩽f⩽2.2f_{r}$ exhibits three main stages. In stage one the bubble shows enhanced non-linearity by which P2 oscillations are generated at very low pressure thresholds. The P2 oscillations disappear by increasing the pressure amplitude however, they re-emerge as P2 or P3 oscillations above a pressure threshold higher than the uncoated counterpart, and in a similar fashion to the uncoated bubble sonicated by its ${\mathrm{Pdf}}_{\mathrm{sh}}$ or $f=2.8-3f_{r}$.

The bifurcation structure of the bubbles when $f=2.8f_{r}$ is shown in Fig. 15e–f. Right at $P_{a}=1\;\mathrm{kPa}$, the bubble with $\sigma_{0}=0$ N/m starts P3 oscillations. The enhanced non-linearity of P3 at such a low excitation is reported for the first time. Pressure amplitude increase leads to a sudden chaos at 104 kPa, with the P3 attractor coexisting with chaos until its disappearance at 112 kPa. Chaos stretches to 156 kPa. Chaotic oscillations become P2 through a cascade of reverse PD bifurcations.

Cases of $f=3f_{r}$ & $\sigma_{0}=0$ N/m and $f=3.1f_{r}$ & $\sigma_{0}=0.072$ N/m are shown in Fig. 15g & h respectively (case of $f=3f_{r}$ and $\sigma_{0}=0.072$ N/m exhibits the similar dynamic as of Fig. 11h. Thus, here we decided to present $f=3.1f_{r}$ to highlight the generation of P3 at $P_{a}=1\;\mathrm{kPa}$). P3 oscillations start at $P_{a}=1\;\mathrm{kPa}$ for both cases. For $\sigma_{0}=0$ N/m, sudden chaos appear at 240 kPa. With pressure amplitude increase P2 oscillations emerge out of the chaotic window through a cascade of reverse PD bifurcations. Lastly P1 oscillations appear above 400 kPa. For $\sigma_{0}=0.072$ N/m, P4 oscillations emerge out of the P3 oscillations through a SN bifurcation and undergo reverse PD bifurcation to P2 and then P1.

### 3.5 Bifurcation structure of the coated bubble with $\sigma_{0}=0.01$ & $\sigma_{0}=0.062$ N/m

Bifurcation structures in this section are also plotted at multiples and fractions of the resonance frequency. Similar to the previous section, the resonance frequency is set to be the frequency of maximum response at 10 kPa. For the bubble with $\sigma_{0}=0.01\;N/m,f_{r}=f_{10\;\mathrm{kPa}}=0.52f_{1\;\mathrm{kPa}}$ & for the bubble with $\sigma_{0}=0.062\;N/m,f_{r}=f_{10\;\mathrm{kPa}}=0.53f_{1\;\mathrm{kPa}}$.

**Fig. 16:**
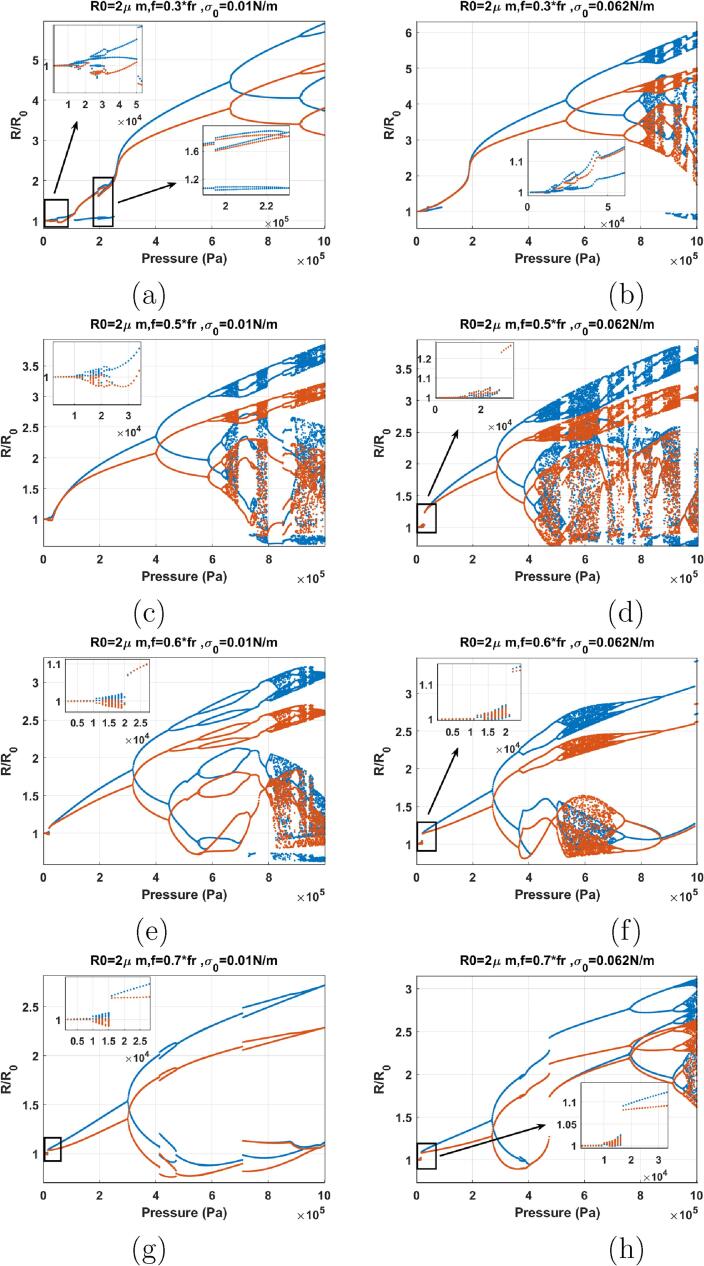
Bifurcation structure of the $R/R_{0}$ of the C3F8 coated bubble with $R_{0}=2\;\mu m$ as a function of pressure amplitude (left: $\sigma_{0}=0.01\;N/m$ and right: $\sigma_{0}=0.062\;N/m$): a–b) $f=0.3f_{r}$, c–d) $f=0.5f_{r}$, e–f) $f=0.6f_{r}$, g–h) $f=0.7f_{r}$.

The bifurcation structures of the bubbles with $\sigma_{0}=0.01$ N/m & $\sigma_{0}=0.062$ N/m insonified by $0.3f_{r}⩽f⩽0.7f_{r}$ are shown in Fig. 16. Fig. 16a–b shows the cases of sonication with $f=0.3f_{r}$. The dynamics of the bubbles are very similar to their counterparts with $\sigma_{0}=0$ N/m & 0.072 N/m sonicated with $f=0.3-0.5f_{r}$ (Fig. 13a–d). However, there are two differences: 1) $\frac{R_{\max}}{R_{0}}$ is generally lower than the initially buckled or the ruptured bubble over all pressures studied and, 2) The threshold for the start of SuH oscillations is ≈11kPa which was 1 kPa in (Fig. 13a–d). The pressure threshold for SuH oscillations is still lower than the case of uncoated bubble in Fig. 11a–b.

Sonication with $f=0.5f_{r}$ is depicted in Fig. 16c–d. There is a general similarity with the bubbles with $\sigma_{0}=0$ & $\sigma_{0}=0.072$ N/m sonicated with $f=0.6f_{r}$ & $f=0.7f_{r}$. Above a pressure threshold in all cases there is a SN bifurcation or an increase in the growth rate of the bubble oscillation amplitude (manifested in the form of an inflection point) which corresponds to the ${\mathrm{Pdf}}_{r}$. At this point the red and blue curve meet indicating the wall velocity with the acoustic excitation is in phase. Referring to Fig. 3, the rate of the decrease of $f_{r}$ with pressure amplitude increase is higher for the bubbles with $\sigma_{0}=0.1$ & 0.072 N/m compared to $\sigma_{0}=0$ & $\sigma_{0}=0.072$ N/m. This manifests itself in the occurrence of the SN or the inflection point at lower frequencies and lower pressures in Fig. 16e–h.

When $f=0.5f_{r}$ the bubbles exhibit 2nd order SuH (P1- 2 maxima) and 5/2 UHs within the pressure amplitude range of 10–28 kPa. Above 28 kPa, the bubble with $\sigma_{0}=0.062$ N/m undergoes a SN bifurcation from a P1 oscillation to another P1 oscillation with higher amplitude. At 57 kPa, the growth rate of the oscillations amplitude increases for the bubble with $\sigma_{0}=0.062$ N/m. This indicates the ${\mathrm{Pdf}}_{r}$ point. Further pressure amplitude increase results in PD and chaotic oscillations. The pressure threshold for PD and $\frac{R_{\max}}{R_{0}}$ are smaller than their counter part with $\sigma_{0}=0$ and 0.072 N/m (Fig. 13c–f).

The dynamics of the bubbles with $\sigma_{0}=0.01$ & 0.062 N/m sonicated with $f=0.6f_{r}$ (Fig. 16e–f) are similar to the case of $f=0.5f_{r}$ in Fig. 16c–d. A SN bifurcation takes place at ≈17kPa for both bubbles and the oscillations amplitude increases abruptly (${\mathrm{Pdf}}_{r}$). Just before the occurrence of SN, a small amplitude chaotic window appears. When SN occurs, blue curve and red curve obtain the same value. As pressure amplitude increases oscillation amplitude increases and the two curve diverge. PD occurs at $P_{a}=300$ and 267kPa respectively for $\sigma_{0}=0.01$ and $\sigma_{0}=0.072$ N/m. The bubble with $\sigma_{0}=0.01$ N/m exhibits the transition from P2 → P4 through a PD and P4 → P8 → P4 through a bubbling bifurcation and then chaos with increasing pressure. The bubble with $\sigma_{0}=0.062$ N/m undergoes P4 and chaos through multiple PDs which is followed by the emergence of P2 oscillations through multiple reverse PD bifurcations out of chaos.

The case of sonication with $f=0.7f_{r}$ is shown in Fig. 16g–h. There are two SN bifurcations with pressure amplitude increase. The initial SN occurs at ≈ 15 kPa and results in P1 oscillations of higher amplitude. After the first SN oscillation amplitude grows with increasing pressure amplitude and PD occurs in both cases. A small P4 window is generated within the P2 window. For the case of $\sigma_{0}=0.01$ N/m and at $P_{a}=710\;\mathrm{kPa}$ P4 oscillations are regenerated and then transition to P2 via reverse PD at 982 kPa. For the bubble with $\sigma_{0}=0.062$ N/m at $P_{a}=479\;\mathrm{kPa}$ P2 oscillations undergo a SN bifurcation to P2 oscillations with higher amplitudes. This is similar to the dynamics of the uncoated bubble sonicated with its ${\mathrm{Pdf}}_{\mathrm{sh}}$ (Fig. 12a–b).

**Fig. 17:**
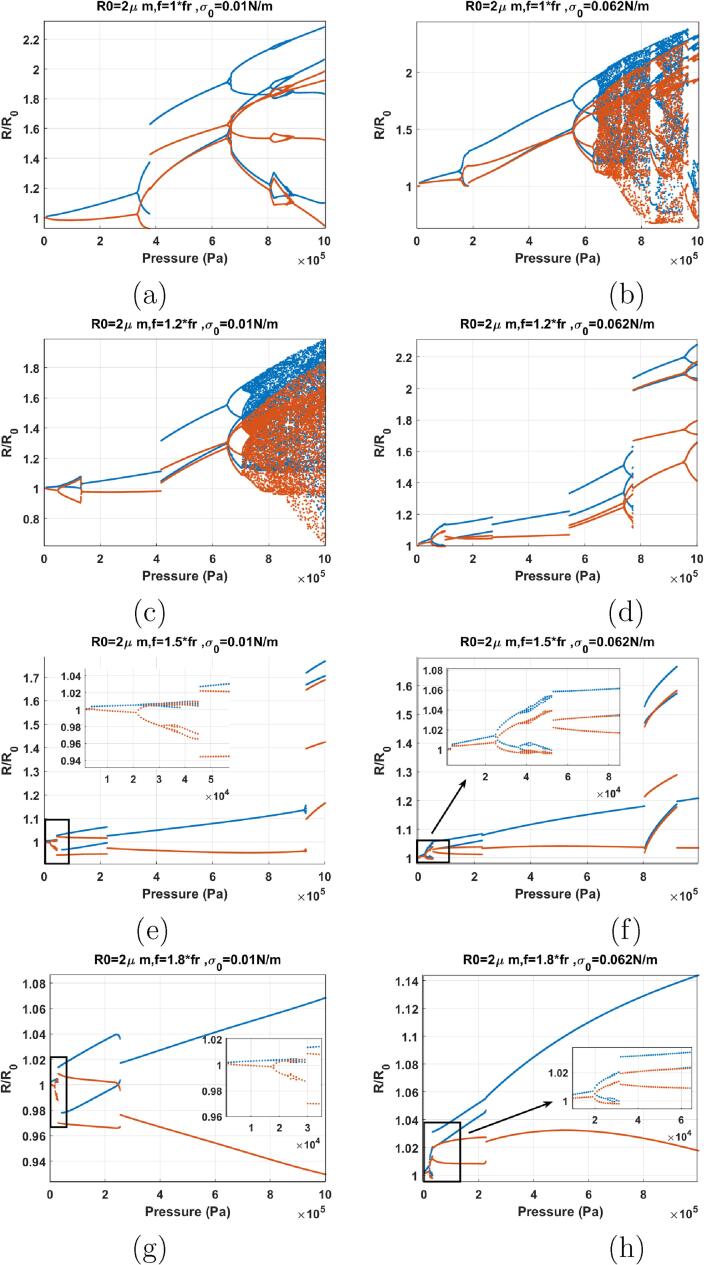
Bifurcation structure of the $R/R_{0}$ of the C3F8 coated bubble with $R_{0}=2\;\mu m$ as a function of pressure amplitude (left: $\sigma_{0}=0.01\;N/m$ and right: $\sigma_{0}=0.062\;N/m$) sonicated with: a–b) $f=f_{r}$, c–d) $f=1.2f_{r}$, e–f) $f=1.5f_{r}$, g–h) $f=1.8f_{r}$.

Case of the $f=f_{r}$ is shown in Fig. 17a-b. At $P_{a}=10\;\mathrm{kPa}$ a SN bifurcation takes place and oscillation amplitudes increase slightly (${\mathrm{Pdf}}_{r}$ at 10 kPa). Oscillation amplitude increases slowly with pressure amplitude and PD occurs at $P_{a}=326\;\mathrm{kPa}$ & 148kPa respectively for $\sigma_{0}=0.01$ N/m & $\sigma_{0}=0.062$ N/m. After the SN, the dynamics of the bubble with $\sigma_{0}=0.01$ N/m (Fig. 17a) & $\sigma_{0}=0.062$ N/m (Fig. 17b) sonicated with $f=f_{r}$ are respectively similar to the dynamics of the bubble with $\sigma_{0}=0$ N/m (Fig. 14e) & $\sigma_{0}=0.072$ N/m (Fig. 14f) sonicated with $f=1.5f_{r}$. For the bubble with $\sigma_{0}=0.01$ N/m increasing pressure amplitude results in a SN bifurcation from P2 oscillations to a higher amplitude P2 oscillations at 378 kPa. This is similar to the dynamics of the uncoated bubble sonicated with its ${\mathrm{Pdf}}_{\mathrm{sh}}$ (Fig. 12a) [58]. P2 oscillations then grow in amplitude with pressure amplitude increase and oscillations become P4-2 through a PD at 624 kPa. Bubble continues with P4 oscillations with a P8 window within, which is created and annihilated through a bubbling bifurcation. The dynamics of the bubble with $\sigma_{0}=0.062$ N/m resembles the case of the uncoated bubble sonicated with $f=2f_{r}$ (Fig. 12c) [34]. P2 oscillations spread between 148-555kPa. At 555 kPa, P4-2 oscillations are generated via a PD and later undergo successive PDs to chaotic oscillations at 638 kPa.

The case for $f=1.2f_{r}$ is shown in Fig. 17c–d. In both cases we witness the generation of the P2 oscillations, their disappearance and re-generation which is similar to the dynamics of the initially buckled and ruptured bubble in Fig. 14g–h. For bubbles with $\sigma_{0}=0.01$ N/m (Fig. 17c) & $\sigma_{0}=0.062$ N/m (Fig. 17d) P2 oscillations occur via a PD at $P_{a}=47$ & 48kPa respectively. With pressure amplitude increase P2 oscillations transition to P1 (disappearance of 1/2 order SHs) at $P_{a}=140$ & 269kPa respectively. P2 oscillations are then re-appear at $P_{a}=418$ & 545kPa respectively. Dynamics of the coated bubbles in this pressure amplitude region is similar to the dynamics of the uncoated bubble sonicated with its ${\mathrm{Pdf}}_{\mathrm{sh}}$ (Fig. 12b). In case of $\sigma_{0}=0.01$ N/m, further pressure amplitude increase results in a cascade of PDs to chaos. In case of the $\sigma_{0}=0.062$ N/m further pressure amplitude increase results in the appearance of P3 oscillations which later undergo PD to P6 oscillations.

The dynamic variation of the effective surface tension due to the lipid coating decreased the frequency of ${\mathrm{Pdf}}_{\mathrm{sh}}$ to frequencies close to resonance. Moreover, P3 oscillations are unexpectedly generated. Compared to the uncoated bubble, the pressure thresholds for P2 oscillations are smaller. Also, $\frac{R_{\max}}{R_{0}}$ are generally smaller than both the uncoated bubble and the bubbles with $\sigma_{0}=0.0$ N/m & $\sigma_{0}=0.072$ N/m.

When $f=1.5f_{r}$ (Fig. 17e–f), PD occurs at $P_{a}=16$ & 21kPa for $\sigma_{0}=0.01$ N/m & $\sigma_{0}=0.062$ N/m respectively and they stretch up to approximately 224 kPa where they transition to P1 oscillations via a SN bifurcation. Further pressure amplitude increase results in the generation of P3 oscillations via another SN bifurcation at $P_{a}=834\;\mathrm{kPa}$ & $P_{a}=805\;\mathrm{kPa}$ respectively for $\sigma_{0}=0.01$ N/m & $\sigma_{0}=0.062$ N/m. The dynamics of the bubble in this region is similar to the dynamics of the uncoated bubble sonicated with $f=2.8-3f_{r}$ (Fig. 12e–f).

For $f=1.8f_{r}$ (Fig. 17g–h), P2 oscillations occur via a PD at $P_{a}=14$ & 18 *kPa*, respectively for $\sigma_{0}=0.01$ N/m & $\sigma_{0}=0.062$ N/m. At 30 kPa P2 oscillations undergo a SN bifurcation to P2 oscillations of higher amplitude. At 255 kPa, P2 oscillations transition to P1 oscillations via another SN. The bubble oscillates with P1 for the rest of the studied pressure amplitude range. In this case the dynamic variation of the effective surface tension of the lipid coating enhances the generation of P2 oscillations at very low pressures. The coating lowers the pressure threshold for the ${\mathrm{Pdf}}_{\mathrm{sh}}$; however, at higher pressures suppresses the nonlinear oscillations.

**Fig. 18:**
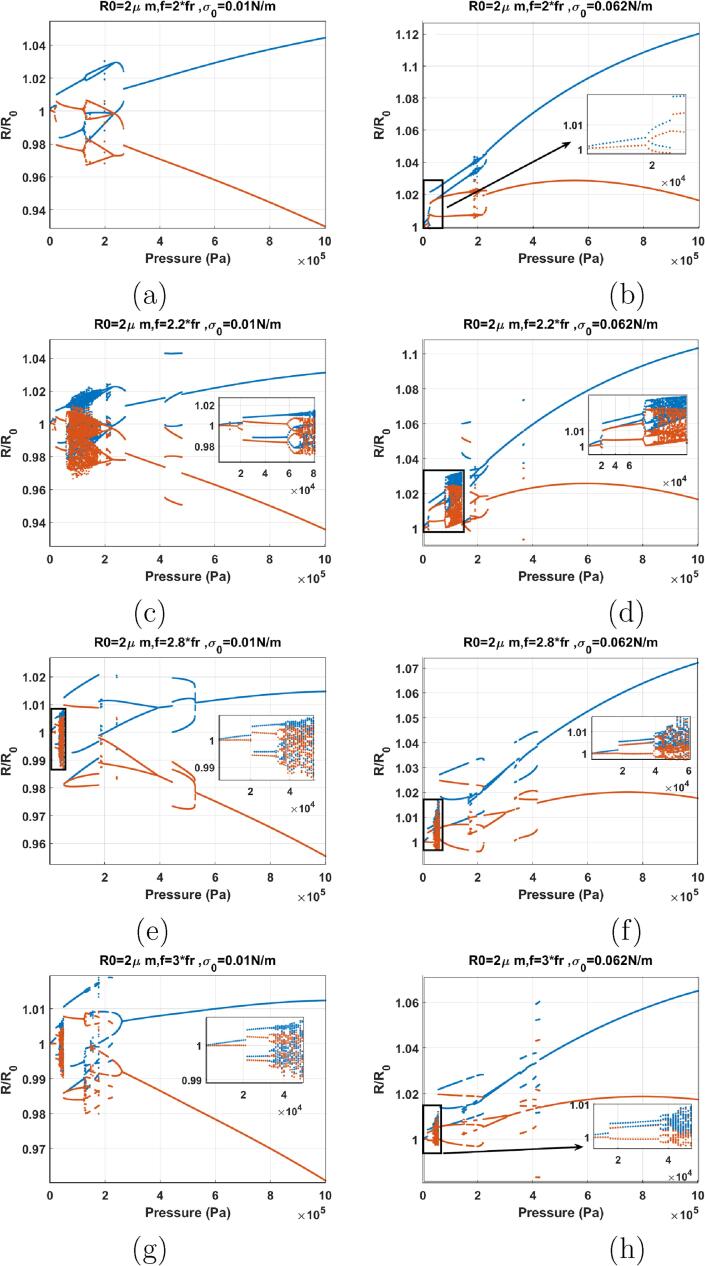
Bifurcation structure of the $R/R_{0}$ of the C3F8 coated bubble with $R_{0}=2\;\mu m$ as a function of pressure amplitude (left: $\sigma_{0}=0.01\;N/m$ and right: $\sigma_{0}=0.062\;N/m$) sonicated with: a–b) $f=2f_{r}$, c–d) $f=2.2f_{r}$, e–f) $f=2.8f_{r}$, g–h) $f=3f_{r}$.

For $f=2f_{r}$ (Fig. 18a–b), PD is initiated at $P_{a}=17\;\mathrm{kPa}$ for both bubbles. A SN bifurcations transition the P2 oscillations to P2 oscillations of higher amplitude at 26 kPa. P4 oscillations are generated and transition back to P2 oscillations through bubbling bifurcation. The P2 oscillations undergo SN bifurcation to P1 oscillations at $P_{a}=268\;\mathrm{kPa}$ & 230kPa respectively for the bubbles with $\sigma_{0}=0.01$ N/m & $\sigma_{0}=0.062$ N/m. Oscillations of the bubble stay at P1 for the rest of the pressure amplitude ranges that is studied here. Compared to the uncoated bubble in Fig. 12c the lipid coating enhances the P2 oscillations at low acoustic pressures; however, the P2 oscillations of the bubble is suppressed at higher pressures.

When $f=2.2f_{r}$ (Fig. 18c–d), P2 oscillations are generated through a PD at $P_{a}=17\;\mathrm{kPa}$ and then at $P_{a}=20\;\mathrm{kPa}$ undergo a SN bifurcation to P2 oscillations of higher amplitude. Oscillations undergo a cascade of PDs to P4 and finally chaos at 71 and 95 kPa for the bubbles with $\sigma_{0}=0.01$ N/m & $\sigma_{0}=0.062$ N/m respectively. For the bubble with $\sigma_{0}=0.01$ N/m and through a cascade of reverse PDs starting at 175 kPa, bubble oscillations transition to a P2 regime, which later undergo a SN bifurcation to P1 at 274 kPa. For the bubble with $\sigma_{0}=0.062$ N/m, the transition from chaos to P2 is via a SN bifurcation at 141 kPa. Oscillations transition to P4 via a SN at 173 kPa followed by reverse PD to P2 and SN to P1 at 236 kPa. P3 oscillations are generated via a SN bifurcation for a small window of pressure amplitude for both bubbles.

When $f=2.8f_{r}$ (Fig. 18e–f), P2 oscillations are generated via SN bifurcations at $P_{a}\approx 20\;\mathrm{kPa}$. Chaos sets in for a small pressure amplitude window of ≈ (39–51 kPa) & (42–59 kPa) for the bubbles with $\sigma_{0}=0.01$ N/m & $\sigma_{0}=0.062$ N/m respectively. P3 oscillations emerge out of the chaotic window via a SN bifurcation and then transition to P2 oscillations via another SN bifurcation at 179 kPa and 229 kPa for the bubbles with $\sigma_{0}=0.01$ N/m & $\sigma_{0}=0.062$ N/m, respectively. For the bubble with $\sigma_{0}=0.01$ N/m, P3 oscillations are re-generated through SN bifurcation at 447kPa and undergo a reverse period tripling at $P_{a}=530\;\mathrm{kPa}$ to P1 oscillations. For the bubble with $\sigma_{0}=0.062$ N/m P3 oscillations are generated at 334 kPa via a SN and then transition again to P1 oscillations via another SN at 416 kPa. The oscillations remain P1 for the rest of the studied pressure amplitude range. When compared to the uncoated bubble, the lipid coating enhanced the generation of P2 and P3 oscillations at lower pressures. The coating also, enhanced the onset of chaos at very low excitation amplitudes and suppressed the chaotic oscillations at higher pressures.

Fig. 18g–h represent the case of $f=3f_{r}$. The dynamics at low pressures $P_{a}<200\;\mathrm{kPa}$ are similar to those in Fig. 18e–f. P2 oscillations are generated at low pressures through a SN which then undergo a cascade of PDs to chaotic oscillations. P3 oscillations then emerge out of the chaotic window through a SN at 49 and 55 kPa, respectively for bubbles with $\sigma_{0}=0.01$ & $\sigma_{0}=0.062$ N/m. For the bubble with $\sigma_{0}=0.01$ N/m, the pressure amplitude increase results in P6 oscillations via a PD at 124 kPa. At 144 kPa, P6 oscillations transition to P2 oscillations via a SN. P2 transition to P6 via another SN at 164 kPa. At 178 kPa, P6 transition to P2 via another SN. At 213 kPa, P2 becomes P3 via a SN which is then followed by a SN from P3 to P2 and reverse PD to P1 for the rest of the studied pressure amplitude range. For the bubble with $\sigma_{0}=0.062$ N/m within the pressure amplitude range of 140–376 kPa, there are intermittent transitions between P2 and P3 via SNs. At 401 kPa, P1 oscillations give birth to a P4 oscillations which then transition to P3 via a SN at 411 kPa. P1 oscillations emerge out of the P3 window via a SN at 425 kPa. Compared to the uncoated bubble, lipid coating enhances the P2, P3 and chaotic oscillations at very low acoustic pressures. Moreover, P4 oscillations appear at $3f_{r}$. In case of the uncoated bubble and for the same initial conditions however, P4 is expected to appear at frequencies near $4f_{r}$ [38], [40].

Compared to the case of $\sigma_{0}=0$ N/m & $\sigma_{0}=0.072$ N/m, P2 and P3 oscillations are not generated right at the 1 kPa driving pressure amplitude and need pressures above 10 kPa. Moreover, the bubbles generally have lower $\frac{R_{\max}}{R_{0}}$.

### 3.6 Bifurcation structure of the coated bubble with $\sigma_{0}=0.036$ N/m

**Fig. 19:**
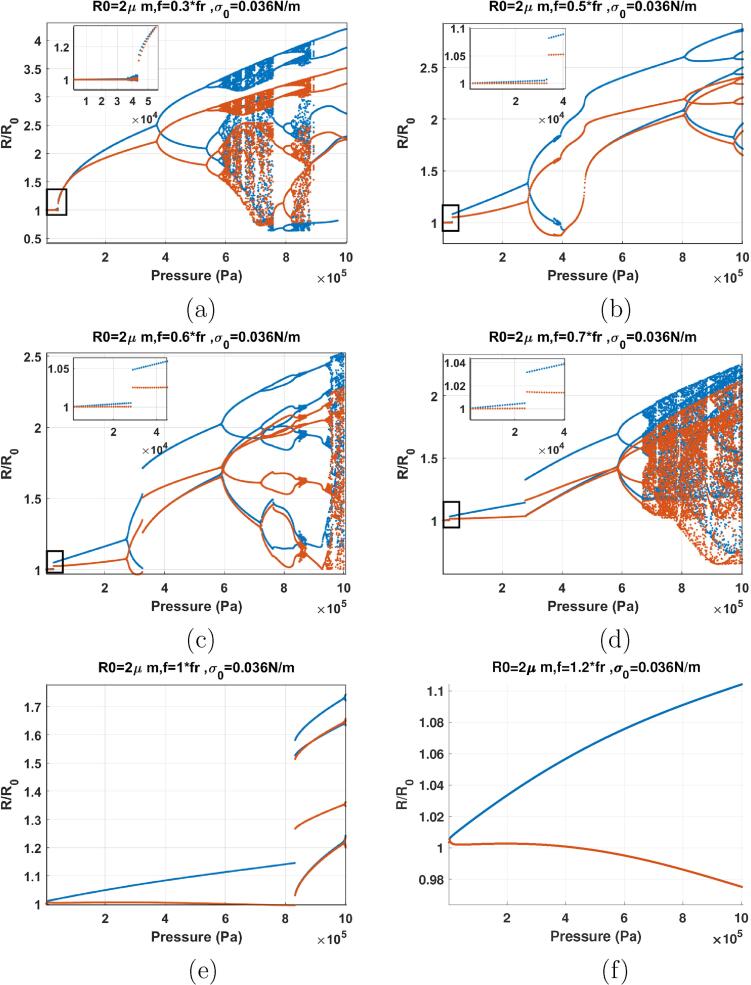
Bifurcation structure of the $R/R_{0}$ of the C3F8 coated bubble with $R_{0}=2\;\mu m$ and $\sigma_{0}=0.036\;N/m$ as a function of pressure amplitude when sonicated with: a) $f=0.3f_{r}$, b) $f=0.5f_{r}$, c) $f=0.8f_{r}$, d) $f=0.9f_{r}$, e) $f=f_{r}$ & f) $f=1.2f_{r}$.

In this section the bifurcation structure of the bubble with $\sigma_{0}=0.036$ N/m is presented. This surface tension is chosen as it is the mid value between the surface tension for buckling and rupture. For the bubble with $\sigma_{0}=0.036N/m,f_{r}=f_{10\mathrm{kPa}}=0.824f_{1\mathrm{kPa}}$. Bifurcation structure of the bubble with $\sigma_{0}=0.036$ N/m is shown in Fig. 19, Fig. 12.

Fig. 19a shows the case of sonication with $f=0.3f_{r}$. P1 oscillations undergo a SN at $P_{a}=44\;\mathrm{kPa}$ and the bubble oscillations amplitude increases abruptly (This is similar to the dynamics of the bubble sonicated with its ${\mathrm{Pdf}}_{r}$ in Fig. 11c-d). Wall velocities are in phase (blue curve meets the red curve) with the driving acoustic pressure for a range of acoustic excitation pressures and with increasing pressure amplitude the two curves diverge. PD occurs at 371 kPa followed by a cascade of PDs leading to chaos at ≈595kPa. Further pressure amplitude increase results in the intermittent transition between chaos and periodic behavior. This behavior is similar to the dynamics of the uncoated bubble sonicated with its pressure dependent resonance frequency (${\mathrm{Pdf}}_{r}$) in Fig. 11a–d. The presence of the coating thus lowers the pressure threshold for the SN bifurcation. However, the pressure threshold for PD is higher and the bubble oscillation amplitude is generally smaller than the uncoated bubble.

When $f=0.5f_{r}$ (Fig. 19b) the bubble undergoes two bifurcations that leads to two abrupt increases in the bubble oscillation amplitude. The first is a SN which takes place at 34 kPa transitioning the P1 oscillations to a P1 with higher amplitude. The second one is an inflection point at 460 kPa transitioning the P2 oscillations to P2 oscillations with slightly higher amplitude. Here, the system exhibits dynamics that are similar to two different regimes of the oscillations in the uncoated bubble. The low pressure amplitude transition is similar to the low pressure amplitude transition of the uncoated bubble sonicated with ${\mathrm{Pdf}}_{r}$ (Fig. 11c–d). The second transition that occurs at a higher pressure amplitude resembles the dynamics of the bubble sonicated with its ${\mathrm{Pdf}}_{\mathrm{sh}}$ in Fig. 12b. When compared to the uncoated counterpart, for the coated bubble the first transition occurs at a lower pressure amplitude while the second transition occurs at a higher pressure.

When $f=0.6f_{r}$ (Fig. 19c), we witness the same two pressure thresholds as the previous case. Two SN occur, one at $P_{a}=29\;\mathrm{kPa}$ and the second one at 327kPa. The first SN transition P1 to a P1 oscillation of higher amplitude (${\mathrm{Pdf}}_{r}$) while the second SN transition the P2 oscillations to P2 oscillations of higher amplitude (${\mathrm{Pdf}}_{\mathrm{sh}}$). Further pressure amplitude increases leads to P4 with bubbles of P8 inside. Right after the bubble 4 small windows of chaos appear which transition to P4 and then again to chaos.

When $f=0.7f_{r}$ (Fig. 19d) two SN takes place; the first SN transitions a P1 oscillation to a P1 oscillation of higher amplitude at 25 kPa (${\mathrm{Pdf}}_{r}$) and the second SN transition the P1 oscillation to P2 oscillations of higher amplitude at 277 kPa (${\mathrm{Pdf}}_{\mathrm{sh}}$). Pressure amplitude increase leads to P4 through a PD at ≈600kPa and later chaos at 671 kPa.

Looking at Fig. 19a–d, the dynamic variation of the surface tension of the lipid coating significantly decreases the frequencies of pressure dependent resonance (${\mathrm{Pdf}}_{r}$) & and pressure dependent SH resonance frequency (${\mathrm{Pdf}}_{\mathrm{sh}}$). As an instance, ${\mathrm{Pdf}}_{\mathrm{sh}}$ typically occurs for $1/5f_{r}<f<2f_{r}$ for the uncoated bubble ([58] and Fig. 12a–b) while here ${\mathrm{Pdf}}_{\mathrm{sh}}$ occurred at frequencies as low as $0.5f_{r}$ for the coated bubble with $\sigma_{0}=0.036$ N/m.

When $f=f_{r}$ (Fig. 19e), an unexpected behavior is observed. P1 oscillations undergo a SN to P3 oscillations at 833 kPa. In case of the uncoated bubble (Fig. 12e–f) or bubbles with pure viscoelastic coating [38], this behavior only occurs for frequencies close to $3f_{r}$. Thus the lipid coating here, decreased the P3 resonance frequency by 200 %. The pressure threshold for P3 oscillations, however is higher for the coated bubble when compared to the uncoated counterpart.

When $f=1.2f_{r}$ (Fig. 19f), nonlinear oscillations are suppressed to only a P1 oscillation for the studied pressure amplitude range.

**Fig. 20:**
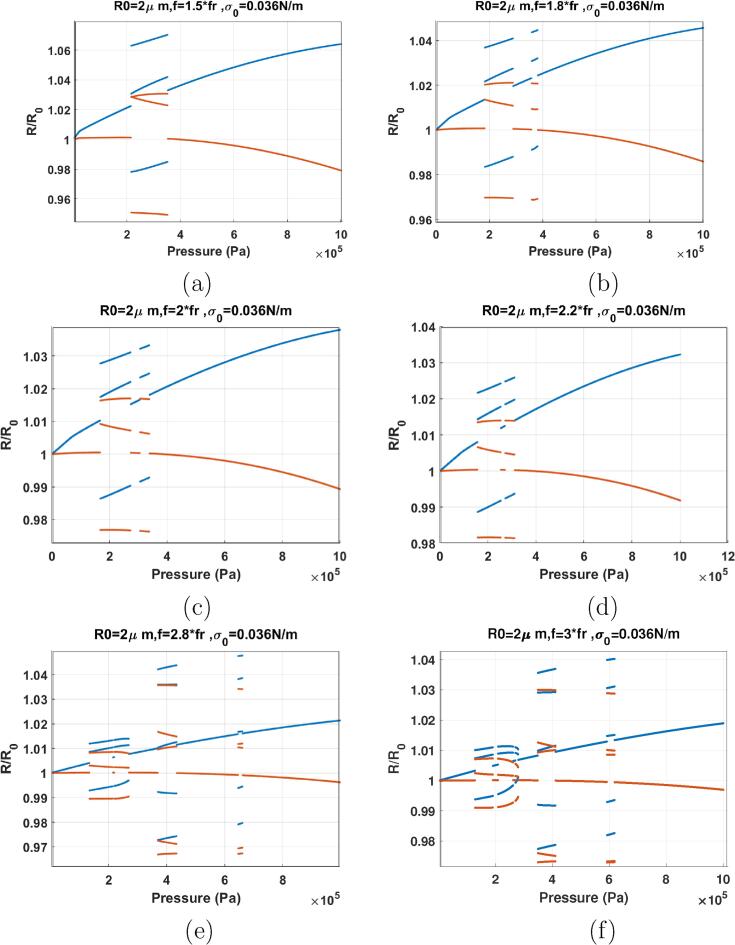
Bifurcation structure of the $R/R_{0}$ of the C3F8 coated bubble with $R_{0}=2\;\mu m$ and $\sigma_{0}=0.036\;N/m$ as a function of pressure amplitude when: a) $f=1.5f_{r}$, b) $f=1.8f_{r}$, c) $f=2f_{r}$, d) $f=2.4f_{r}$, e) $f=2.8f_{r}$ & f) $f=3f_{r}$.

For $f=1.5f_{r}-2.2f_{r}$ (Fig. 20a–d), P3 oscillations are enhanced. Compared to the P3 oscillations in case of the uncoated bubble (Fig. 12e–f), P3 occurs at lower pressure thresholds. For instance at $f=2.2f_{r}$ P3 is generated at 157 kPa. This is however, higher than the pressure threshold for P3 oscillations in case of the coated bubbles with $\sigma_{0}=0,0.01,0.62$ & 0.072 N/m.

When $f=2.8f_{r}$ (Fig. 20e), P3 is generated through a SN at 134 kPa and later transition to P1 via another SN at 269 kPa. P5 oscillations are generated at 373 kPa through a SN and transition to P1 at 433 kPa. P5 oscillations re-appear again for a short pressure window through the same mechanism at 647 kPa.

When $f=3f_{r}$ (Fig. 20f), P3 oscillations start at 128 kPa and stretch up to 279 kPa with a short window of P1 oscillations within. P5 oscillations are generated at 351 and 592 kPa for two short pressure windows. Compared to the uncoated bubble sonicated with $3f_{r}$ (Fig. 12f), the pressure threshold of P3 oscillations is lowered by about 276 %.

Coating with $\sigma_{0}=0.036$ N/m significantly reduced the frequency for P3 and P5 oscillations. Most interestingly, the coated bubble with $\sigma_{0}=0.036$ N/m exhibits enhanced P3 oscillations over a very large frequency range of $f_{r}⩽f⩽3f_{r}$.

### 3.7 Investigation of the mechanism of the disappearance (standstill) and regeneration of P2

**Fig. 21:**
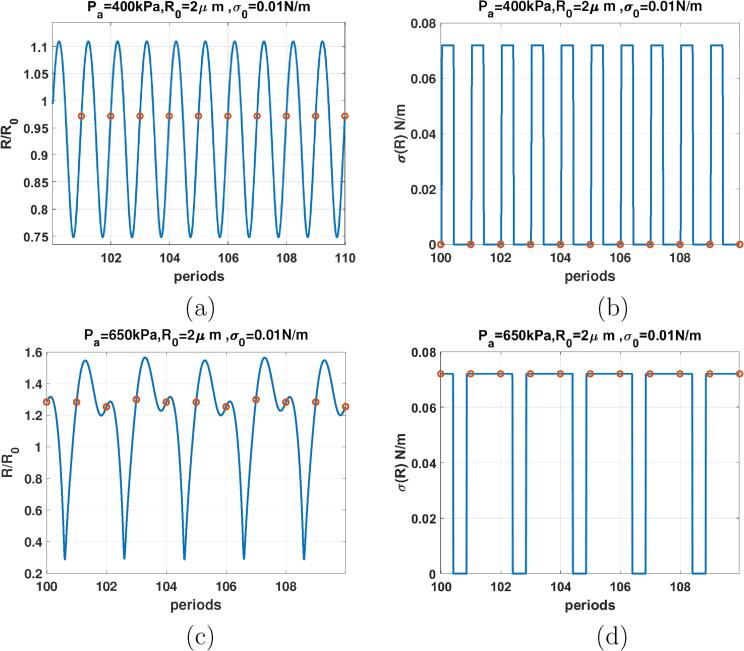
$R/R_{0}$ (left) & σ(R) (right) as function of the driving periods for a C3F8 coated bubble with $R_{0}=2\;\mu m$ with $\sigma_{0}=0.062$ N/m when $f=1.2f_{r}$ for: a & b-$P_{a}=400\;\mathrm{kPa}$, c& d-$P_{a}=650\;\mathrm{kPa}$. (Red circles correspond to the location of R(t) at each period). (For interpretation of the references to colour in this figure legend, the reader is referred to the web version of this article.)

In subSection 3.2 we showed that the enhancement of P2 oscillations at lower pressures can be caused by the asymmetric variations of the effective surface tension due to buckling or rupture. Here, we look into the possible reasons of the disappearance of the P2 oscillations when increasing pressure. Fig. 21a shows the radial oscillations as a function of the driving periods of the coated bubble in Fig. 17d ($R_{0}=2\mu m$ and $\sigma_{0}=0.062$ N/m) at $P_{a}=400\;\mathrm{kPa}$. At this pressure amplitude the P2 oscillation regime disappeared. Radial oscillations are P1, and the red circles return only one value. The corresponding σ(R) curve, depicts a rather symmetrical variations in the buckling and rupture, the bubble spends the same approximate time in the buckled stage as the ruptured stage. For 10 driving periods, the bubble buckles 10 times and ruptures 10 times.

As pressure amplitude increases, P2 is regenerated (Fig. 17d). At 650 kPa the radial oscillations vs period curves have two maxima (Fig. 21)c and the red circles have 2 distinct values. σ(R) as a function of the driving periods (Fig. 21d) exhibits an asymmetrical behavior between the buckled and the ruptured state. The bubble spends a longer time duration at the ruptured stage than the buckled stage. As a result, the bubble buckles 5 times and ruptures 5 times within 10 driving periods. Thus oscillations become P2 again.

## 4 Summary of the results and discussion

### 4.1 Sonication with $f<f_{r}$

First the findings related to the sonications with frequencies smaller than resonance are presented.

#### 4.1.1 $\sigma_{0}=0,0.01,0.062$ and 0.072 N/m

1- SuH regimes are generated at lower excitation thresholds compared to the uncoated bubbles. The bubbles initially at the buckled or the ruptured stages exhibit SuH regime of oscillations at the lowest pressure threshold of 1 kPa. Thus applications of coated bubbles with initial surface tension close to 0 N/m or 0.072 N/m have the potential to increase the contrast in super harmonic imaging (e.g. [95]). Due to the lower threshold of the SuH generation, the amplitude of the generated harmonics in tissue will be smaller. Therefore, the contrast to tissue ratio may be higher.

2- The sudden increase in the bubble oscillation amplitude (SN bifurcation or the inflection point in bifurcation diagrams) occurs at lower excitation amplitudes when compared to the uncoated bubble and coated bubbles with linear viscoelastic behavior [56]. The SN bifurcation is more pronounced in case of the bubbles with $\sigma_{0}=0.01$ N/m and $\sigma_{0}=0.062$ N/m. The wall velocity stays in phase with the driving sound field for a larger pressure amplitude range. The reason for the lower pressure threshold for the SN and lower frequencies of ${\mathrm{Pdf}}_{r}$ is the fast decrease of the resonance frequency with increasing pressure. Overvelde et al. [68] has experimentally and numerically shown that for coated microbubbles undergoing buckling, the nonlinear resonance behavior is enhanced at pressures as low as 10 kPa. Helfield and Goertz [77] experimentally observed the enhanced nonlinear resonance behavior of the lipid coated microbubbles at pressures of 13–25 kPa. The SN bifurcation can have applications in amplitude modulation techniques [96].

3- For the coated bubble with $\sigma_{0}=0$ N/m, PD occurs at a higher pressure threshold compared to the uncoated bubble, and for other cases, PD occurs at lower pressure thresholds.

4- For $0.6f_{r}⩽f⩽0.7f_{r}$ and for coated bubbles with $\sigma_{0}=0.01$ & 0.062 N/m, P2 oscillations (with resonant 3/2 order UHs) are generated within non-destructive regimes of oscillations $R_{\max}/R_{0}<2$. For the uncoated bubble and coated bubbles with $\sigma_{0}=0$ & 0.072 N/m, PD most likely results in bubble destruction. In [34], [58] we have shown that in case of uncoated bubbles PD may be concomitant with bubble destruction when the bubble is sonicated with $f⩽f_{r}$. The stabilizing effect of the coating with $\sigma_{0}=0.01$ & 0.062 N/m can enhance the non-destructive PD for the frequencies below resonance.

5- In case of coated bubbles with $\sigma_{0}=0.01$ & $0.062N/m,{\mathrm{Pdf}}_{\mathrm{sh}}$ can occur at frequencies as low as $0.6f_{r}$. In such cases two SN bifurcations are observed with increasing pressure. The first SN occurs at a lower pressure threshold and transfers a P1 oscillation to a P1 oscillation of higher amplitude. The second SN occurs at a higher pressure amplitude and transfers a P2 oscillation to a P2 oscillation of higher amplitude. In [58] we have shown that ${\mathrm{Pdf}}_{\mathrm{sh}}$ typically occurs at $1.5f_{r}<f<2f_{r}$ and can lead to oversaturation of the 1/2 order SH component of the scattered signal. The enhanced nonlinear resonance behavior of the coating thus shifts the ${\mathrm{Pdf}}_{\mathrm{sh}}$ to frequencies below resonance. The occurrence of the two SNs may have potential applications in increasing the contrast in multi-pulse amplitude modulation techniques.

#### 4.1.2 Case of the coated bubble with $\sigma_{0}=0.036$ N/m

1- Compared to the uncoated bubble and coated bubbles with linear viscoelastic behavior (${\mathrm{Pdf}}_{r}$ is within $0.5f_{r}<f<f_{r}$ [56]), the frequency of ${\mathrm{Pdf}}_{r}$ is much lower (as low as $0.3f_{r}$).2- ${\mathrm{Pdf}}_{\mathrm{sh}}$ can occur even at $f=0.5f_{r}$. In case of the uncoated bubble ${\mathrm{Pdf}}_{\mathrm{sh}}$ occurs at $1.5f_{r}<f<2f_{r}$ [58].3- For $0.5f_{r}⩽f\leq 0.7f_{r}$ and with increasing pressure, two SN occur; the first one transfers a P1 oscillation regime to a higher amplitude P1 and the second one which is at a higher pressure transfers a P1 or a P2 oscillation regime to a higher amplitude P2 regime.

### 4.2 $f⩾f_{r}$

In this section findings of the sonications with frequencies above resonance are summarized. Such a frequency range is typically used in SH imaging of microbubbles in contrast enhanced ultrasound [20], [68], [78], [96].

#### 4.2.1 Cases of the coated bubbles wiht $\sigma_{0}=0,0.01,0.062$ and 0.072 N/m

1- For the coated bubbles with $\sigma_{0}=0$ N/m & $\sigma_{0}=0.072$ N/m sonicated with $f_{r}⩽f⩽1.5f_{r}$ and for the ones with $\sigma_{0}=0.01$ N/m & $\sigma_{0}=0.062$ N/m sonicated with $f=f_{r}$, the bifurcation structure is similar to the case of sonication with ${\mathrm{Pdf}}_{\mathrm{sh}}$ in case of the uncoated bubbles. P2 oscillations undergo a SN from a P2 oscillation to a P2 oscillation of higher amplitude. The nonlinear behavior of the coating thus reduces the ${\mathrm{Pdf}}_{\mathrm{sh}}$ to frequencies below $1.5f_{r}$. Thus for the coated bubbles with $\sigma_{0}=0$ N/m & $\sigma_{0}=0.072$ N/m, sonication with $f_{r}⩽f⩽1.5f_{r}$ may result in a stronger 1/2 order SH component of the scattered signal because of the over-saturation that takes place when $f={\mathrm{Pdf}}_{\mathrm{sh}}$.

2- For the coated bubbles with $\sigma_{0}=0$ N/m & $\sigma_{0}=0.072$ N/m that are insonated with $1.8f_{r}⩽f⩽2.2f_{r}$, with increasing pressure, P2 oscillations are generated through a PD (at a pressure threshold of 1 kPa), disappear and then are regenerated at a higher pressure amplitude as a higher amplitude P2 or P3. The second P2 is similar to the dynamics of the uncoated bubble undergoing a SN to P2 when $f={\mathrm{Pdf}}_{\mathrm{sh}}$. The second P3 is similar to the dynamics of the uncoated bubble undergoing a SN to P3 when $f\approx 3f_{r}$. In [73], the disappearance of SH oscillations is referred to as an “unexpected standstill” of SHs. This means that, in the case of a bubble able to generate a stable subharmonic oscillation, the subharmonic emission disappears if the acoustic pressure is raised above a second pressure threshold. The subharmonic standstill however, is a reversible [73]; that is, if the acoustic pressure amplitude is decreased again, the bubbles start generating subharmonics one more time[73]. Thus, disappearance is not due to the bubble destruction. Prior works on subharmonics performed on a population of microbubbles did not report this kind of behavior because it was probably ‘masked’ by the overall response of the several other bubbles within the same sample volume that experience different pressure amplitudes [73]. The standstill of subharmonic emission also was not explained by the numerical studies of the nonlinear models of the bubble dynamics. Here, we show that the disappearance of the SHs is due to the symmetric buckling and rupture of the shell at moderate pressures. At higher pressures, similar to the lower pressures the buckling and rupture of the shell becomes asymmetric. This manifests itself in the re-generation of P2 signals. Above the second pressure threshold, the bubble spends more time in the ruptured stage than the buckling stage. This exposes the bare gas to water for a longer duration and thus can explain the reduced stability of SH oscillations when they were re-generated [73].

In sensitive therapeutic applications like blood–brain barrier opening, the SH components of the scattered pressure by microbubbles are commonly used as a signature for quantifying the nonlinear oscillations of the bubble cloud and treatment efficacy [97], [98]. Due to the strong interplay between stable and inertial cavitation regimes, understanding the origin and stability of P2 oscillation regimes is crucial. Thus, the information on the generation, disappearance, amplification and stability of the P2 oscillations that is obtained here, provides a framework for the analysis of the optimization of SH oscillations in applications.

3- For the coated bubbles with $\sigma_{0}=0.01$ N/m & $\sigma_{0}=0.062$ N/m sonicated with $f=1.5f_{r}$, with increasing pressure, P2 oscillations are generated through a PD and then disappear via a SN. Above a second pressure threshold, a P3 oscillation regime occurs via a SN from a P1 regime. This is similar to the dynamics of the uncoated bubble undergoing a SN to P3 when $f\approx 3f_{r}$. The pressure threshold for PD is smaller than the uncoated bubbles [40] and the coated bubbles with linear viscoelastic behavior [38].

4- For the coated bubbles with $\sigma_{0}=0.01$ N/m & $\sigma_{0}=0.062$ N/m sonicated with $1.8f_{r}⩽f⩽2f_{r}$, with increasing pressure, P2 oscillations are generated through a PD and then are amplified via a SN. P2 oscillations are then transfer to a P1 regime via another SN. Bubble oscillations remain P1 for the rest of the pressure amplitude range studied in this paper.

5- For the coated bubbles with $\sigma_{0}=0$ N/m & $\sigma_{0}=0.072$ N/m and for $2.8f_{r}⩽f⩽3.1f_{r}$, P3 may occurs at very low pressure amplitudes (as low as 1 kPa). Chaos can emerge at pressures lower than 200 kPa.

6- The lowest pressure threshold for the chaotic oscillations are for the coated bubbles with $\sigma_{0}=0.01$ N/m & $\sigma_{0}=0.062$ N/m when sonicated with $2.8f_{r}⩽f⩽3f_{r}$ which is followed by the emergence of P3 out of the chaotic window. To our best knowledge, such low pressure thresholds for chaotic oscillations has not been observed in a bubble oscillator. The pressure threshold for P3 is approximately 5 times smaller than the uncoated counterpart.

Here we identified several different types of SN that occur with increasing pressure amplitude in the oscillations of the lipid coated bubbles. This information, can provide the fundamental framework for the optimization of amplitude modulation techniques and SH imaging procedures. Moreover, the enhanced P3 and higher order oscillations may find potential in mixing applications and drug delivery.

In the cases analyzed in this paper, $\frac{R_{\max}}{R_{0}}$ was higher for the bubbles with a higher $\sigma_{0}$ because of the expansion dominated behavior of the bubble. This can be one of the reasons for the lower pressure threshold of P2 and chaotic oscillations in case of the bubble in the ruptured state.

#### 4.2.2 Case of the coated bubble with $\sigma_{0}=0.036$ N/m

1- For $1.5f_{r}⩽f⩽3f_{r}$ with increasing pressure amplitude a P3 occurs via a SN through a P1 oscillation regime. The pressure threshold for P3 is about half of the uncoated counterpart. P3 disappears via a SN. A second or 3rd SN may occur with pressure increase that can lead to the regeneration of P3 or the generation of P5 or P7 oscillations. Due to the wide range of the pressure amplitude and frequency of P3 behavior for the bubbles with $\sigma_{0}=0.036$ N/m, engineering of the coatings with such initial surface tensions may find potential in higher order SH imaging with potential higher resolution and contrast. In [59] we have shown that the 2/3 or 3/4 order SHs are stronger than 1/2 order SHs and due to their close proximity to the transducer central frequency they may be detected with superior sensitivity.

## 5 Limitations and future work

The goal of this paper was to study the influence of the lipid coating on the nonlinear dynamics of the MBs. Thus for simplicity we only analyzed the radial oscillations of the bubble. Future work can be extended by analyzing the scattered pressure of the bubbles to find the regions of SH power enhancement. Bubble–bubble interaction should also be considered as in applications bubbles exist in poly-disperse clouds. The bifurcation structure of the interacting bubbles has been studied extensively in [99], [100], [101], [102], [103], [104], [105]. These studies have shown that the bubble–bubble interaction significantly influences the dynamics of each bubble. Effects of coupling, bubble size, and spatial arrangement have been studied in [102] and effects of boundary proximity on the dynamics of a bubble cluster is investigated in [103]. We have shown in [105] that the bubble cluster may exhibit collective behavior dominated by the response of the larger bubbles. Future studies need to look into the effect of the interaction of lipid coated MBs and potential collective behavior of the lipid coated bubbles at lower excitation pressure amplitudes.

This study was limited to the case of sonication with a single frequency acoustic excitation. Dynamics of MBs that are sonicated with dual-frequency acoustic excitation have been investigated in several studies [47], [49], [50], [52], [51], [55], [106], [107], [108]. Sonication with two frequency forcing can be used to suppress chaos [47], [49], [50] or enhance the nonlinearity of the system [47], [49], [50], [52], [51], [55]. Dual frequency forcing may also be used to enhance the bubble expansion to achieve a higher chemical yield [106], [107]. The dynamics of lipid coated MBs excited by multi-frequency acoustic excitations can be a subject of future studies. The enhanced non-linearity of such a system may find potential new applications and improvements in contrast enhanced imaging and therapy.

In this study non-spherical oscillations of the MBs were also neglected. Pioneering work of Holt and Crum [109] investigated the subharmonic behavior of larger bubbles (≈100-200μm in size) and have experimentally observed the shape oscillations concomitant with subharmonic oscillations. They showed that the appearance of shape oscillations could be phenomenologically mistaken for a simple period-doubling of the radial mode. Versluis et al. [110] through using high speed optical observations were able to identify shape oscillations of mode n = 2 to 6 in the behavior of single air bubbles with radii between 10μm and 45 μm. Their study [111] showed that the close to resonance bubbles were found to be most susceptible toward shape instabilities. In case of coated MBs, non-spherical bubble oscillations were investigated in [111] through high speed optical observations. It was shown that non-spherical bubble oscillations are significantly present in medically relevant ranges of MB radii and applied acoustic pressures. Non-spherical oscillations develop preferentially at the resonance radius and may exist during SH oscillations [111]. Recently Klapcsik and Hegedüs[46] through GPU accelerated large parameter investigations and 2D bifurcation diagrams, have shown that non-spherical oscillations can affect the subharmonic threshold and nonlinear behavior of bubbles. Most recently Guédra et al. [112] through optical observations have shown that subharmonic oscillations can be triggered by energy transfer from surface to volume oscillations and thus can change the pressure threshold for SH emissions. Thus, for a more accurate modeling of the nonlinear behavior of lipid coated MBs, deeper theoretical modeling of MB coating, accounting for membrane shear and bending is required [111].

One of the limitations of the current study is neglecting the effects of lipid shedding and mass transfer. These effects may become important at higher pressures and they should be considered for accurate modeling. The lipid coating undergoes buckling and rupture when the bubble oscillates. The lipid coating reseals quickly when the bubble contracts [113], [114]. For a long enough pulse and depending on the applied pressure amplitude, the coating may shed some lipids while it undergoes buckling, rupture and reseal [115], [116]. This leads to mass transfer and shrinkage of the bubble which reaches a stable size after a few cycles [114], [115]. The incorporation of these effects can be the subject of future studies.

In this work we studied the bifurcations structure of the bubble oscillator using the standard methods of bifurcation analysis. The buckling and rupture of the shell, however, makes the lipid coated bubble a non-smooth system [117], [118], [119]. Similar features of the bifurcation structure of the lipid coated bubble may be seen in the behavior of the pressure relief valve model which is a non-smooth system. Future studies that focus on the nonlinear properties of the lipid coated bubble, can reveal more detailed information about the system behavior using the tools of non-smooth dynamics [118], [119].

## 6 Conclusion

In this work, the bifurcation structure of the lipid coated bubbles undergoing buckling and rupture was studied extensively. Our results further confirmed that the rapid variation of the effective surface tension and buckling and rupture of the coating enhances the generation of nonlinear behavior including higher order SHs, SuHs and chaos. We showed for the first time that P2 and P3 can occur at pressures as low as 1 kPa (≈1% of the ambient pressure). Existence of chaos was confirmed at pressures as low as 10 kPa. The closer the initial surface tension of the bubble to the buckling stage or the ruptured stage, the lower the pressure threshold for the nonlinear behavior. We showed that rapid variations of the surface tension on the bubble may not be enough for enhanced non-linearity. In case of asymmetrical variations of the surface tension between buckling and rupture, nonlinear behavior is enhanced. However, symmetrical behavior of the effective surface tension may suppress the non-linearity.

## CRediT authorship contribution statement

**A.J. Sojahrood:** Formal analysis, Investigation, Methodology, Project administration, Software, Validation, Visualization, Writing - original draft, Writing - review & editing. **H. Haghi:** Software. **R. Karshafian:** Funding acquisition, Writing - review & editing. **M.C. Kolios:** Funding acquisition, Supervision, Resources, Writing - review & editing.

## Declaration of Competing Interest

The authors declare that they have no known competing financial interests or personal relationships that could have appeared to influence the work reported in this paper.

## Figures and Tables

**Fig. A.figurekk:**
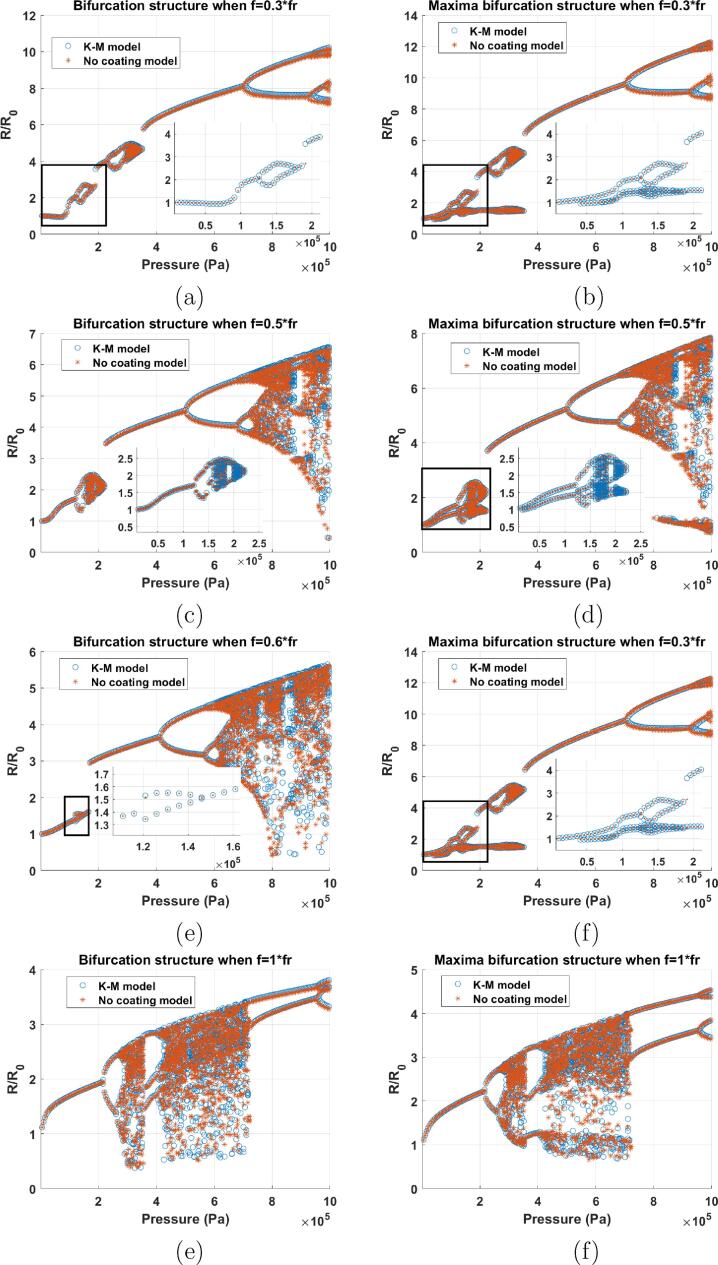
Comparison between the bifurcation structures of the Marmottant model with no shell (Eq. (A.1)) and Keller-Miksis model (Eq. (3)) for a C3F8 gas bubble with $R_{0}=2\;\mu m$ for $f⩽f_{r}$. Left column: Poincaré cross section method (2.3.1), Right column: Method of maxima (2.3.2).

**Fig. A.figurekk:**
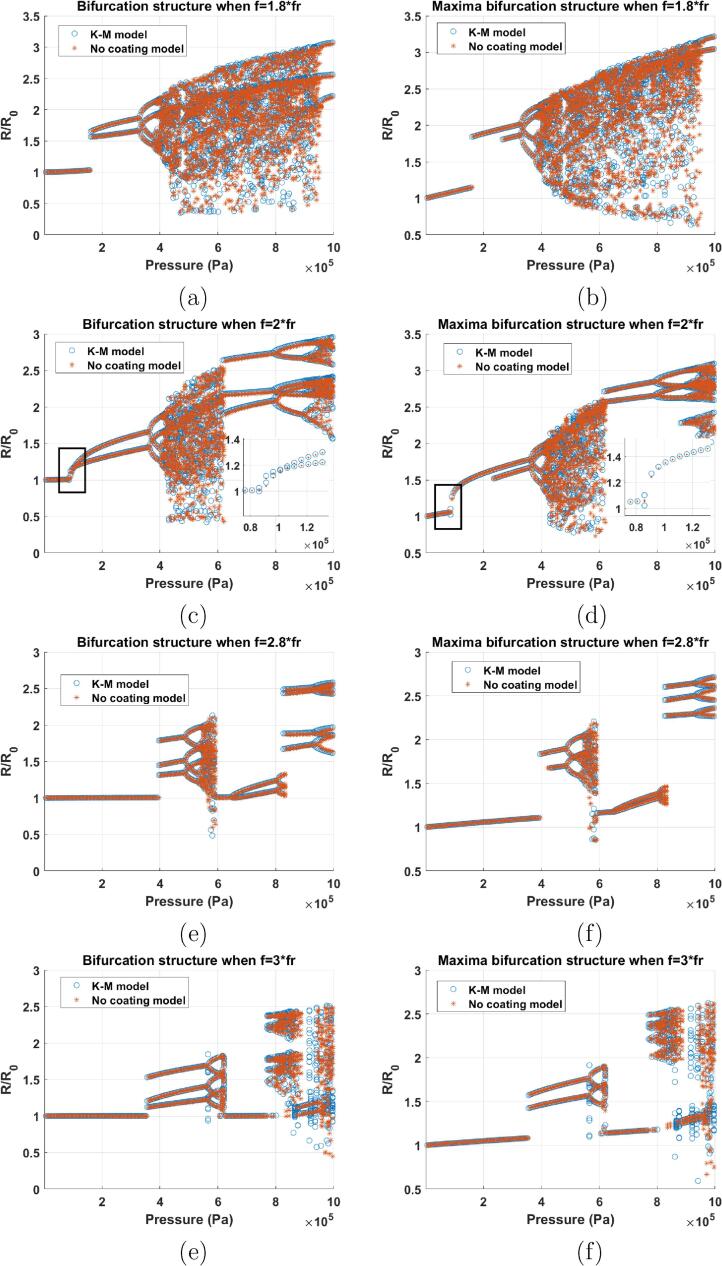
Comparison between the bifurcation structures of the Marmottant model with no shell (Eq. (A.1)) and Keller-Miksis model (Eq. (3)) for a C3F8 gas bubble with $R_{0}=2\;\mu m$ for $\mathrm{fr}<f⩽3f_{r}$. Left column: Poincaré cross section method (2.3.1), Right column: Method of maxima (2.3.2).

**Fig. B.figurekk:**
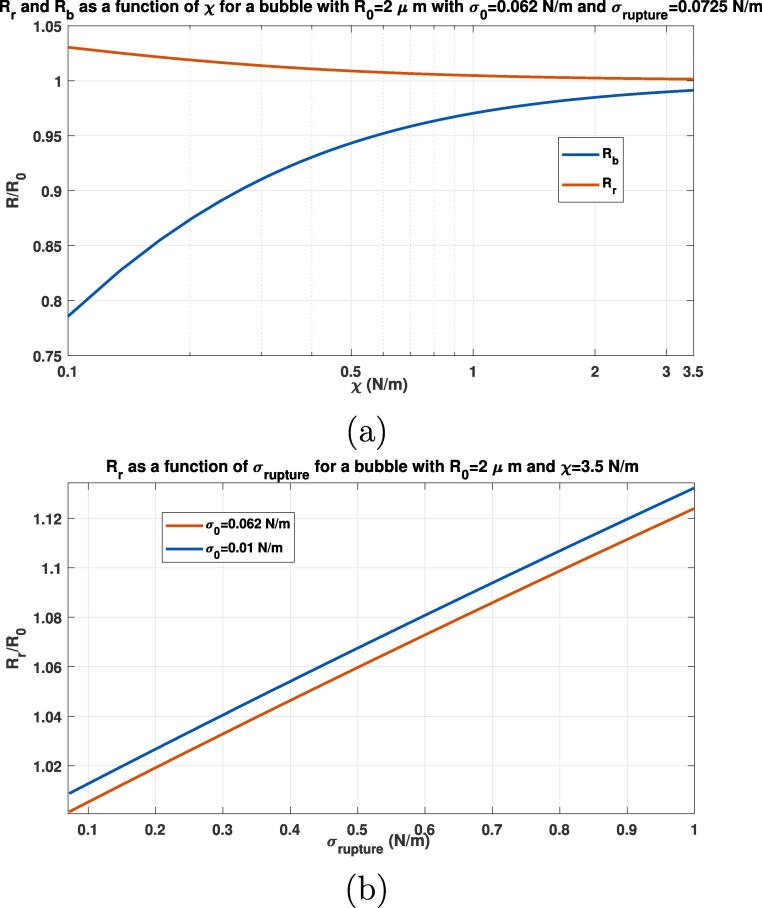
Case of a C3F8 lipid coated bubble with $R_{0}=2\;\mu m$: a) variations of $R_{r}$ and $R_{b}$ as a function of χ when $\sigma_{0}=0.062\;N/m$ and $\sigma_{\mathrm{rupture}}=0.0725\;N/m$, b) Variations of $R_{r}$ as a function of $\sigma_{\mathrm{rupture}}$ when χ=3.5N/m and $\sigma_{0}=0.01\;N/m$ and 0.062N/m.

**Fig. C.figurekk:**
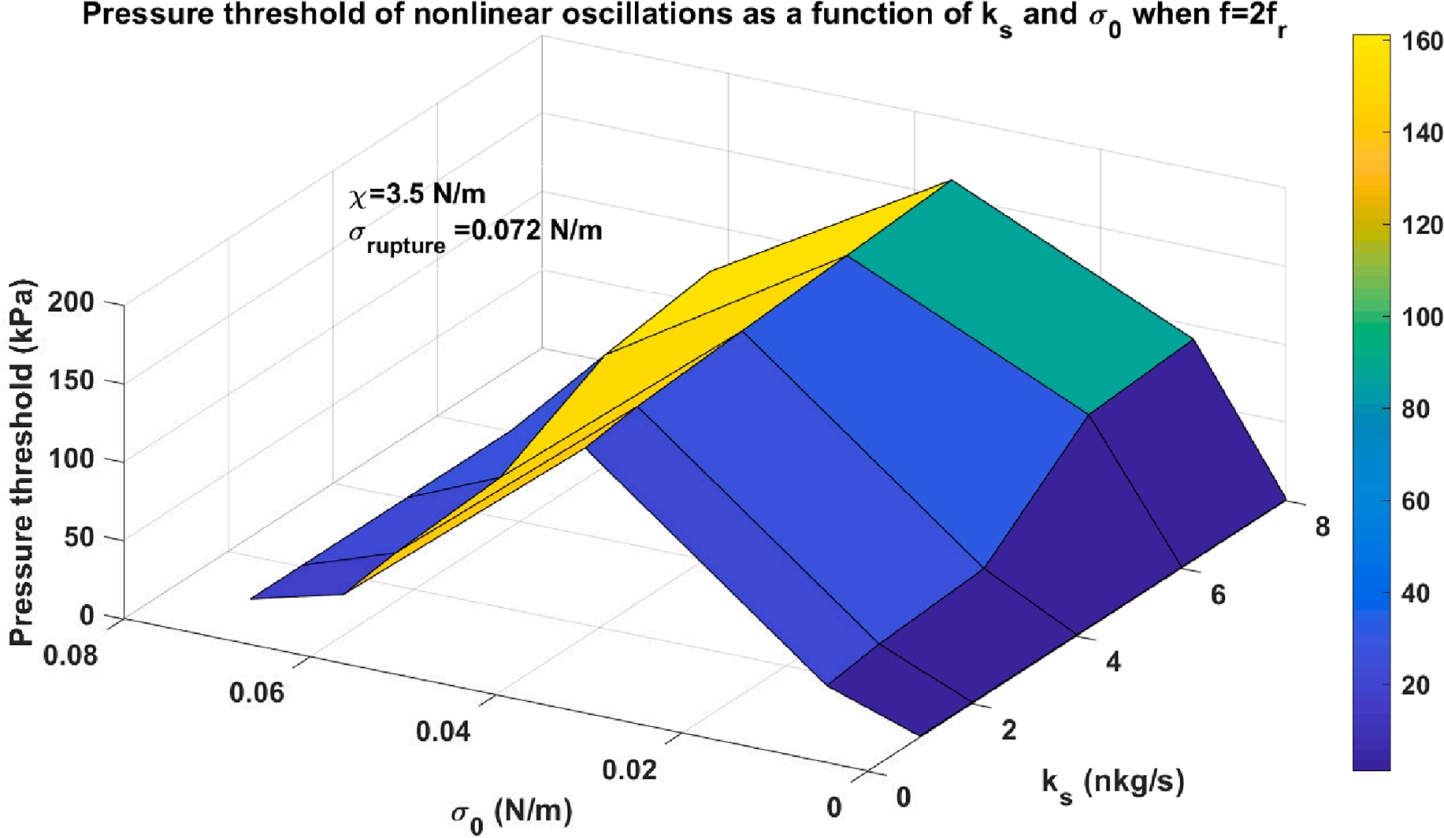
Pressure threshold for the onset of nonlinear oscillations (P2 or P3) as a function of $k_{s}$ and $\sigma_{0}$ when $f=2f_{r}$ for a C3F8 lipid coated bubble with χ=3.5N/m and $\sigma_{\mathrm{rupture}}=0.072$ N/m. The colors and the z-axis represent the pressure threshold for the onset of nonlinearity.

## Appendix A Comparison between the Marmottant model with no coating and the Keller-Miksis model

The Marmottant model [62] in the absence of the coating reduces to:

(6) $$\rho \left(R\overset{¨}{R}+\frac{3}{2}{\overset{˙}{R}}^{2}\right)=\left[P_{0}+\frac{2\sigma }{R_{0}}\right]{\left(\frac{R}{R_{0}}\right)}^{-3k}\left(1-\frac{3k}{c}\overset{˙}{R}\right)-P_{0}-\frac{2\sigma }{R}-\frac{4\mu \overset{˙}{R}}{R^{2}}-P_{a}(t)$$

In this equation some of the terms of the Keller-Miksis (K-M) equation [81] are dropped. This is because K-M model is no longer valid when $\frac{\left|\overset{˙}{R}\right|}{c}\approx 1$. [59]. The Marmottant model is in a popular form that is developed for sonoluminescence and ultrasound contrast agent (UCA) modeling [120], [121]. The results of the Marmottant model were compared to the K-M uncoated bubble model to investigate the shell effects. However, as the forms of the K-M model and the Marmottant model in the absence of coating are not the same, the comparison may not seem justified. In this section we show that the K-M model in Eq. (3) and Eq. (A1) indeed predict the same **overall** behavior over the exposure parameter ranges that were studied in our paper. Thus the comparison between the models is justified. Fig. A.figurekk, Fig. A.figurekk show that the predictions of the bifurcation structure of $R/R_{0}$ vs pressure of the Marmottant model with no shell (Eq. (A.1)) are in good agreement with the Keller-Miksis model (Eq. (3)) within the exposure parameter ranges that were studied in this work. It should be noted that the comparison between the coated bubble model and the free bubble model are qualitative. In other words, the uncoated bubble behavior was used as a reference to reveal the nonlinear effects induced by the presence of the shell. However, we see here that despite the difference in some terms of the two equations, the quantitative behavior of ($R/R_{0}$) of the uncoated bubble as predicted by Eq. (A.1) and Eq. (3) are in agreement. The radial oscillation amplitude and the pressure threshold for the onset of nonlinear behavior (e.g. PD, chaos) are in agreement. However, although both models predict the same pressures for the onset and disappearance of chaos, when the oscillations are chaotic, the radial oscillation amplitudes are not always equal. For better visualizing the predictions of the two models at lower pressure amplitudes, the figure insets concentrate on the lower acoustic pressure regions $P_{A}⩽250\;\mathrm{kPa}$.

## Appendix B Influence of χ and $\sigma_{\mathrm{rupture}}$ on the buckling and rupture radius

Analysis in this paper showed that the nonlinear behavior of the lipid coated MBs is enhanced at very low pressures. This occurs due to the rapid variation of the effective surface tension when the bubble radius exceeds above the rupture radius or contracts below the buckling radius. Fig. B.figurekk.a shows that for a constant $\sigma_{0}$ and $\sigma_{\mathrm{rupture}}$ as χ increases, $R_{r}$ decreases and $R_{b}$ increases ($R_{r}$ and $R_{b}$ converge to $R_{0}$); thus, a lipid MB with a higher χ which is not initially at buckled or ruptured state exhibits nonlinear behavior at a lower pressure threshold.

MBs with resistant shells that can withstand higher tensions before rupture (e.g. with $\sigma_{\mathrm{rupture}}>0.1$ N/m) has been observed by Marmottant et al. [62]. Fig. B.figurekk.b shows that $R_{r}$ increases with increasing $\sigma_{\mathrm{rupture}}$. For a lipid bubble with initial surface tension of more than 0.036 N/m, the pressure threshold for enhanced non-linearity will increase as $\sigma_{\mathrm{rupture}}$ increases. This is because $R_{r}$ increases linearly with $\sigma_{\mathrm{rupture}}$, thus higher pressure is needed to enhance the nonlinear oscillations. A detailed understanding of the influence of χ and $\sigma_{\mathrm{rupture}}$ over a wide pressure and frequency range can be a subject of future investigations.

## Appendix C Influence of $k_{s}$ on the pressure threshold of the onset of nonlinear oscillations

Fig. C.figurekk shows the pressure threshold for the onset of nonlinear oscillations for a C3F8 lipid coated MB with χ=3.5 N/m and $\sigma_{\mathrm{rupture}}=0.072$ N/m as a function of the shell viscosity $k_{s}$ and $\sigma_{0}$. The frequency is $2f_{r}$. The pressure threshold is extracted from the radius vs pressure amplitude bifurcation diagrams for P2 or P3 oscillations. Increasing $k_{s}$ does not change the pressure threshold ($P_{a}=1\;\mathrm{kPa}$) for nonlinear oscillations for the MBs initially at buckled or ruptured stage. For MBs which are not initially at buckled or ruptured stage, the pressure threshold increases with increasing $k_{s}$. As an example for a MB with $\sigma_{0}=0.062$ N/m, the pressure threshold increases from 16 kPa to 71 kPa when $k_{s}$ is increased from $1\;*\;10^{-9}$ to $8\;*\;10^{-9}$ kg/s. Bubbles which are initially at a buckled or ruptured state start nonlinear oscillations at the lowest pressure threshold ($P_{A}=1\;\mathrm{kPa}$), regardless of the value of $k_{s}$.
